# BrrICE1.1 is associated with putrescine synthesis through regulation of the arginine decarboxylase gene in freezing tolerance of turnip (*Brassica rapa* var. *rapa*)

**DOI:** 10.1186/s12870-020-02697-6

**Published:** 2020-11-04

**Authors:** Xin Yin, Yunqiang Yang, Yanqiu Lv, Yan Li, Danni Yang, Yanling Yue, Yongping Yang

**Affiliations:** 1 Key Laboratory for Plant Diversity and Biogeography of East Asia, Kunming Institute of Botany, Chinese Academy of Science, Kunming, 650204 China; 2 Plant Germplasm and Genomics Center, Kunming Institute of Botany, Chinese Academy of Sciences, Kunming, 650201 China; 3 Institute of Tibetan Plateau Research at Kunming, Kunming Institute of Botany, Chinese Academy of Sciences, Kunming, 650201 China; 4 University of Chinese Academy of Sciences, Beijing, 100049 China; 5 Changchun Normal University, Changchun, 130032 China; 6 College of Landscape and Horticulture, Yunnan Agricultural University, Kunming, 650201 China

**Keywords:** *Brassica rapa var. rapa*, Freezing tolerance, Transcriptome, Metabolome, Putrescine

## Abstract

**Background:**

In the agricultural areas of Qinghai-Tibet Plateau, temperature varies widely from day to night during the growing season, which makes the extreme temperature become one of the limiting factors of crop yield. Turnip (*Brassica rapa var. rapa*) is a traditional crop of Tibet grown in the Tibet Plateau, but its molecular and metabolic mechanisms of freezing tolerance are unclear.

**Results:**

Here, based on the changes in transcriptional and metabolic levels of Tibetan turnip under freezing treatment, the expression of the arginine decarboxylase gene *BrrADC2.2* exhibited an accumulative pattern in accordance with putrescine content. Moreover, we demonstrated that BrrICE1.1 (Inducer of CBF Expression 1) could directly bind to the *BrrADC2.2* promoter, activating *BrrADC2.2* to promote the accumulation of putrescine, which was verified by RNAi and overexpression analyses for both *BrrADC2.2* and *BrrICE1.1* using transgenic hair root. The function of putrescine in turnip was further analyzed by exogenous application putrescine and its inhibitor DL-α-(Difluoromethyl) arginine (DFMA) under freezing tolerance. In addition, the BrrICE1.1 was found to be involved in the ICE1-CBF pathway to increase the freezing stress of turnip.

**Conclusions:**

BrrICE1.1 could bind the promoter of *BrrADC2.2* or *CBFs* to participate in freezing tolerance of turnip by transcriptomics and targeted metabolomics analyses. This study revealed the regulatory network of the freezing tolerance process in turnip and increased our understanding of the plateau crops response to extreme environments in Tibet.

## Background

Polyamines are low molecular weight aliphatic polymers that are widely present in various organisms. Many types of polyamines, such as agmatine, putrescine, spermidine, spermine, and cadaverine, have been found in higher plants [1]. Among them, putrescine acts as the first key factor in polyamine and the synthesis of polyamines begin with the synthesis of putrescine metabolism in most organisms. Putrescine in plants could be formed in two ways: arginine decarboxylase (ADC) and Orn decarboxylase (ODC) pathway [2, 3]. The ADC pathway is catalyzed by three enzymes in sequences: ADC, agmatine iminohydrolase (AIH), and *N*-carbamoylputrescine amidohydrolase (CPA). Additionally, spermidine synthetase (SPDS) catalyzes putrescine to produce spermidine, and spermine synthetase (SPMS) catalyzes the combination of spermidine and aminopropyl to produce spermine, in which the aminopropyl is generated from S-adenosylmethionine (SAM) by SAM decarboxylase [3].

Numerous studies have demonstrated that changes and balance in polyamine metabolism have important regulatory effects on the growth and stress response of many plants [4, 5], including that to chilling stress [6], high-temperature stress [7], drought [8], salinity [9], disease [10], and oxidative stress [11]. Increasing endogenous polyamines by genetic transformation of polyamine biosynthetic genes or exogenous application of polyamines enhanced the tolerance in various plants [6, 12, 13]. For example, overexpression of the key genes *ADC1* and *ADC2* for putrescine synthesis in *Arabidopsis thaliana* increased the contents of putrescine, improving cold tolerance, but mutants defective in *adc1* and *adc2* displayed reduced freezing tolerance compared to control plants [8, 14]. Study by (Urano et al. 2003) [9] also showed that *ADC2* was induced under salt stress in *Arabidopsis* and that the content of free putrescine was regulated to enhance the resistance to stress, while the *adc2* mutant was very sensitive to salt stress. In addition, transgenic plants expressing the yeast *SAMDC* showed enhanced tolerance to high-temperature stress [15]. Additionally, exogenous polyamines and polyamine inhibitors are important means to study the relationship between polyamines and plant stress resistance. For example, exogenous putrescine and spermidine could reduce the stress of salt-sensitive rice [16], but polyamine inhibitors (such as DFMA) reduced the cold tolerance of chilling-tolerant rice [17]. Furthermore, exogenous application of putrescine improved potato cold-acclimated freezing tolerance [6]. Therefore, regulation of the polyamine metabolic pathway is an effective way to improve plant resistance to stresses and has broad application prospects in plant breeding for stress resistance.

Freezing stress is a major factor that limits crop yield, preventing crop growth in millions of hectares worldwide and reducing the geographical distribution of crops. Plants have evolved complex mechanisms to adapt to extreme temperatures, such as low-temperature stress, which was accompanied by changes in the expression of thousands of genes [18]. Among them, the ICE1-DREB/CBF axis was identified as a key regulatory pathway under low temperature in plants [19]. ICE1 is a well-characterized helix-loop-helix (bHLH) protein that acts as an upstream regulator of the transcriptional regulation cascade of the cold response in *Arabidopsis*. ICE1 regulates the transcriptional expression of downstream genes by binding to the MYC element (CANNTG) on the promoter of the *CBF* gene, and *CBF* in turn regulates many cold-regulated (*COR*) genes [20–22]. However, the possibility of other unexplored potential mechanisms of *ICE1* cannot be completely eliminated. In fact, in addition to the established ICE1-CBF cascade, the *CdICE1* of chrysanthemum could also mediate freezing resistance by regulating the expression of the microRNA miR398 [23]. In particular, PtICE1 interacted with ADC, which is associated with the polyamine pathway regulating cold tolerance in *Poncirus trifoliata* [24]. Thus, some undetermined molecular mechanisms may also contribute to the cold tolerance of *ICE1* and its homologues.

The Qinghai-Tibet Plateau has a special climate, with a high altitude, a low temperature, low oxygen, strong radiation, drought, strong wind and other adverse climatic conditions occurring frequently. In the long-term adaptation process, plants have evolved many survival strategies. In this regard, understanding how plants in Tibet adapt to and survive abiotic stresses is important for the efficient exploitation of genetic resources associated with desirable stress tolerance, for developing new approaches to enhance stress tolerance, and for providing important theoretical support for the analysis of multiple crops in the plateau. Turnip (*Brassica rapa var. rapa*) is a traditional crop in the Qinghai-Tibet Plateau that is used for food, feed and medicine and has good adaptability to the extreme environment in the long-term growth process of the Qinghai-Tibet Plateau [25]. Research has shown that Tibetan turnip was highly responsive to cold exposure [26]. However, very limited information was available about the mechanism underlying the freezing tolerance of turnip. Analysis of the adaptability of turnip to the freezing environment will help elucidate the molecular mechanism of freezing tolerance of turnip and could also provide suggestions for breeding turnip in Tibet. In present study, through analysis of differential gene expressions and weighted gene coexpression correlation network analysis (WGCNA) by transcriptome and metabonomic studies, we identified a key gene *BrrADC2.2* that may be involved in putrescine metabolism. BrrICE1.1 binding to *BrrADC2.2* promoter regulated putrescine synthesis which were identified by biochemical and *Agrobacterium rhizogenes*-mediated (LBA9402 strain) genetic transformation assays, indicating the important roles of putrescine in freezing stress tolerance. In addition to the classical ICE1-CBF-COR pathway, the polyamine metabolic pathway contributed to Tibetan turnip freezing resistance under extremely low temperature conditions.

## Results

### The Tibetan turnip KTRG-B49 response to freezing tolerance

**Fig. 1:**
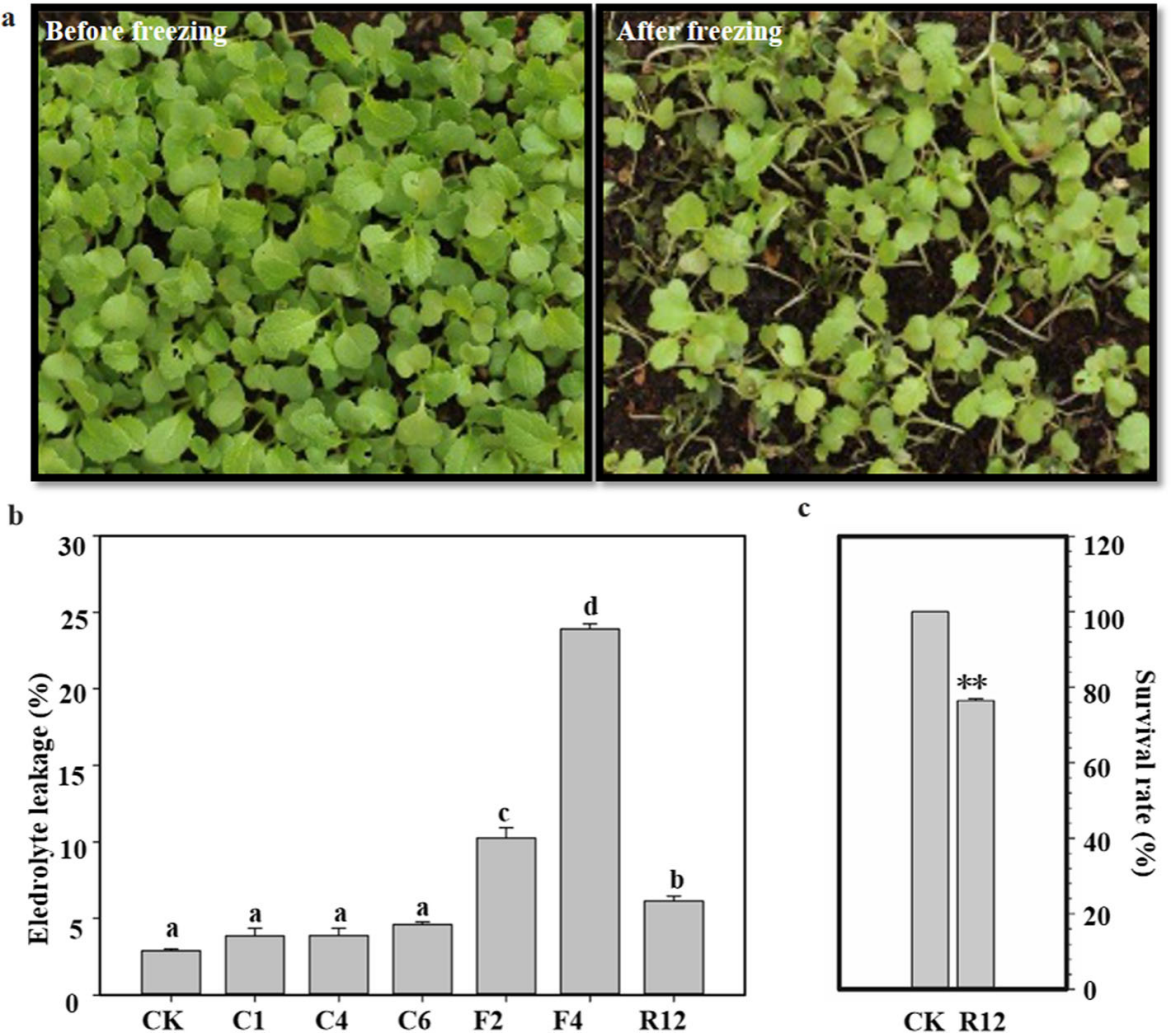
Freezing treatment and physiological index determination of turnip KTRG-B49. **a** Freezing phenotypes. **b** Electrolyte leakage (%). Values are the mean of 5–8 biological replicates. Bars indicate SD. Different symbols indicate significant differences between treatments (*P* < 0.05) according to Tukey’s test. CK, C1, C4, C6, F2, F4, and R12 represent the samples from the untreated control; 4 °C at 1 h, 3 h, and 6 h; − 2 °C at 2 h; − 4 °C 1 h; and recovery for 12 h, respectively. **c** Survival rates. Asterisks indicate statistically significant differences (*P < 0.05, **P < 0.01, Student’s *t* test)

We used the turnip KTRG-B49 variety from the Qinghai-Tibet Plateau to assess the freezing tolerance of turnip. As expected, compared with the control seedlings, most of the treated seedlings survived (Fig. 1a). With the extension of freezing stress time, the electrolyte leakage of the turnip cells increased gradually (Fig. 1b). When the plants were grown at − 4 °C for 1 h, they displayed more serious damage than plants grown under standard conditions. After recovery growth for 12 h in an ambient environment, the plants were restored and had an electrolyte leakage of 23.9%, but there was still a high survival rate of 78.9% (Fig. 1c). These data indicated that KTRG-B49 had the reproductive characteristics of turnip on the Qinghai-Tibet Plateau and can respond to low temperatures.

### Transcriptome changes in KTRG-B49 during freezing treatment

**Fig. 2:**
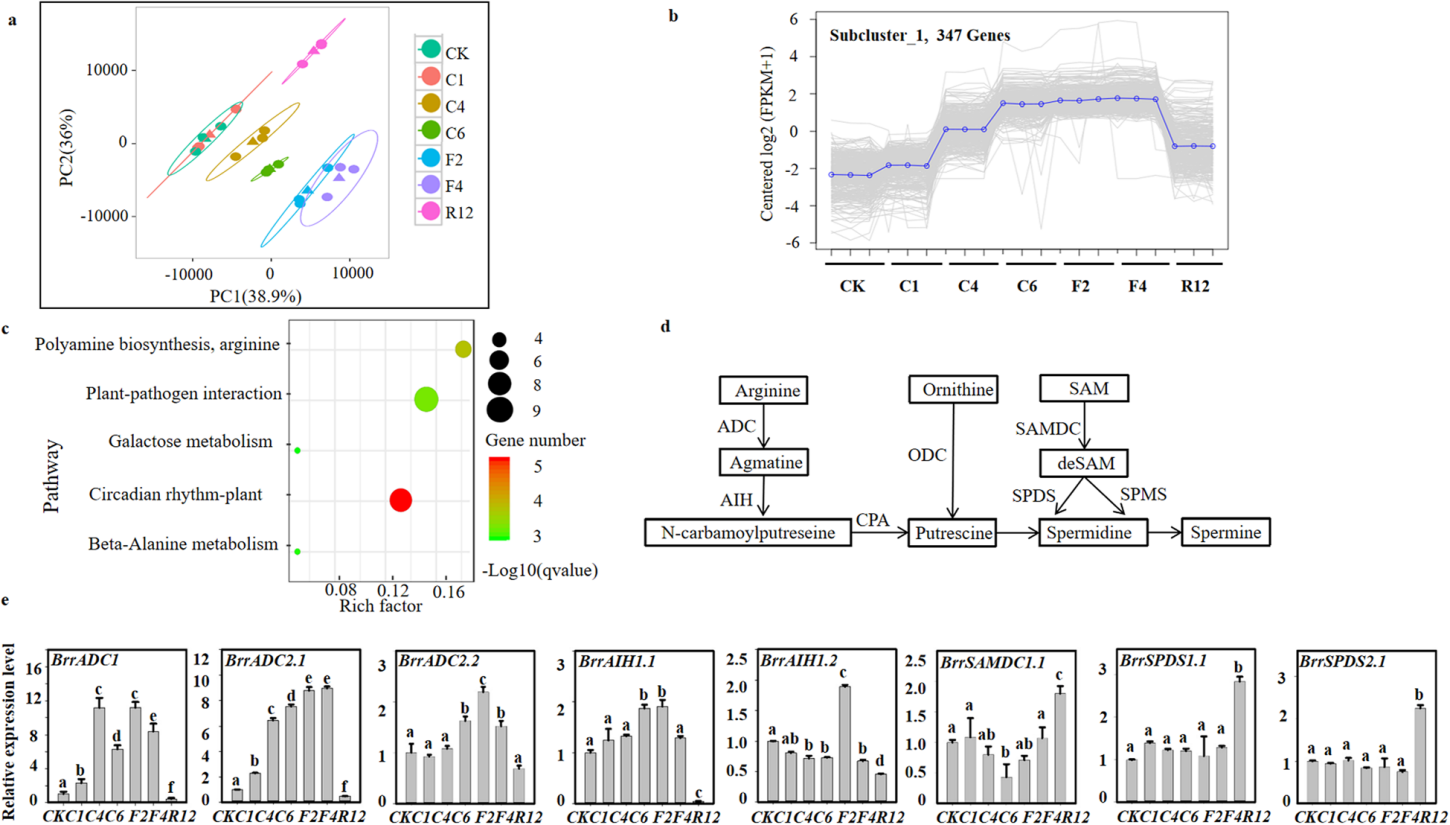
Transcriptome and qRT-PCR analysis of key genes involved in the polyamine biosynthesis pathway in turnip. **a** Principal component analysis (PCA) of transcriptome data. The scale of the axis is the relative distance. Different colors or shapes represent different groups of samples under freezing treatments. **b** Different trend (subcluster_1) analysis of DEGs. **c** Scatterplot of KEGG pathways enriched for differentially expressed genes (DEGs) in subcluster_1. The top 5 enriched pathway terms in the KEGG database are listed. **d** Polyamine biosynthetic pathway (right) in plants. **e** qPCR analysis of key gene in polyamines biosynthesis, with three biological and technical replicates. The data are analyzed by one-way ANOVA (Tukey’s test). Different letters indicate significant difference (*P* < 0.05). CK, C1, C4, C6, F2, F4, and R12 represent the samples from the untreated control; 4 °C at 1 h, 3 h, and 6 h; − 2 °C at 2 h; − 4 °C 1 h; and recovery for 12 h, respectively

KTRG-B49 plants that underwent freezing treatment were used for RNA-seq to determine the genes responsible for tolerance. Totals of 144.16 Gb clean data were obtained for 21 KTRG-B49 individuals, with more than 6.18 Gb for each sample. The sequencing quality scores of 30 (Q30) of the samples were greater than 89.03%, indicating the high quality of the reads (Table S2). Here, 83.27 -89.72% of the clean reads were mapped to the turnip reference genome using TopHat software with an average mapping rate of 86.56% (Table S3). Therefore, more than half of the turnip reference genes were expressed in each sample, which was sufficient for further differential gene expression analysis. A correlation analysis of the expression levels among the samples (Fig. S2) and the FPKM distribution for all samples (Fig. S3) showed that the repeatability of the expression level of each sample was good. In addition, the results of principal component analysis (PCA) based on the 21 samples showed that the first component explained 38.9% of the variation, and the second component explained 36% of the variation (Fig. 2a). KTRG-B49 could be clearly separated five distinct clusters along the first component axis. This finding indicated a close relationship between CK and C1 and between F2 and F4, which may have similar expression patterns. The differential expression analysis among different treatments can be inferred based on PCA.

### The polyamine synthesis pathway is associated with the freezing responses of KTRG-B49

To investigate the gene expression levels, we calculated the FPKM values using the reads from RNA-seq. The number of differentially expressed genes (DEGs) and functional annotation information are shown in Fig. S4 and Table S4, respectively. In the analysis, the DEGs were statistically grouped into six subclusters based on their expression patterns in turnip at different treatment stages (Fig. S5). Interestingly, 347 genes in subcluster_1 demonstrated a similar trend in the PCA of the transcriptomic landscape, which deserves further study (Fig. 2b). Accordingly, KEGG enrichment analysis of the DEGs in subcluster_1 was conducted to identify pathways that played important roles in the freezing process. The top five pathways for the upregulated DEGs are displayed, and major pathways related to circadian rhythm-plant, plant-pathogen interaction, polyamine biosynthesis, and arginine (Fig. 2c), which could indicate their involvement in turnip freezing tolerance. Here, based on these results and the role of polyamine in freezing stress, we focused on the pathway of polyamine synthesis (Fig. 2d). The expression levels of the DEGs in the polyamine pathway were verified by qRT-PCR (Fig. 2e). Of these genes, *BrrADC1, BrrADC2.1*, *BrrADC2.2*, and *BrrAIH1.1* accumulated continuously under freezing stress, showing a similar trend to subcluster_1. These DEGs related to polyamine synthesis may play a special role in enhancing the freezing resistance in turnip.

### Putrescine was specifically enriched in KTRG-B49 upon freezing treatment

**Fig. 3:**
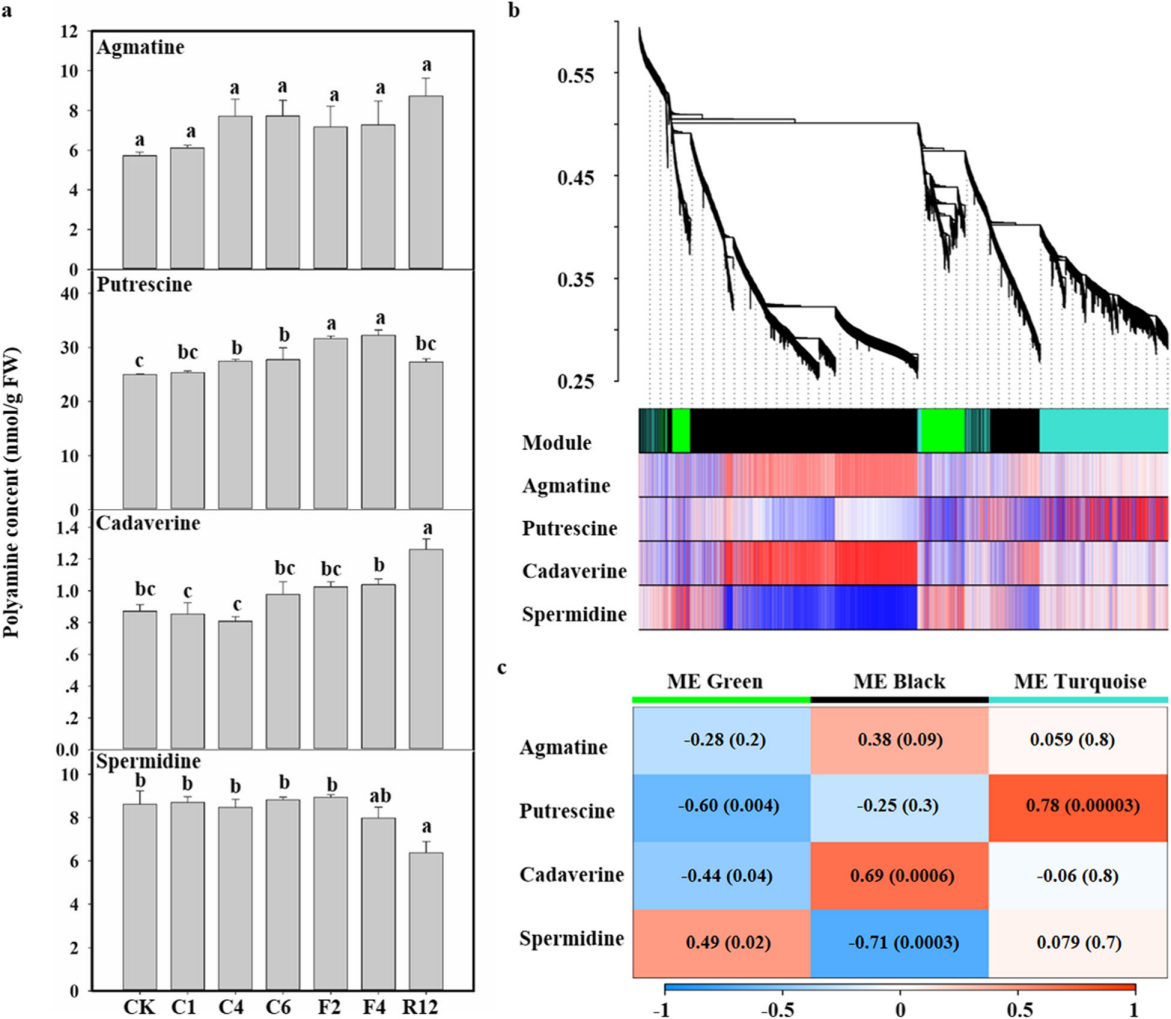
Determination of polyamine content after freezing treatments and weighted gene coexpression network analysis (WGCNA) analysis of module eigengenes and polyamine metabolic profiles. **a** The contents of agmatine, putrescine, cadaverine, and spermidine under freezing treatment in turnip. Values are the mean of five biological replicates. Bars indicate SD. Different symbols indicate significant differences between treatments (*P*  <  0.05) according to Tukey’s test. **b** Clustering dendrogram of expressed genes. Gene modules were identified by dynamic hierarchical tree cut and shown in different colors. **c** For each module, the heatmap showed module eigengene (ME) correlations to traits. Numbers in each rectangular indicate the correlation coefficients and Student’s asymptotic P value for significant ME-trait relationships. The scale bar, bottom, indicates the range of possible correlations from positive (red, 1) to negative (blue, -1)

To confirm that the polyamine pathway was associated with turnip freezing tolerance, we analyzed polyamine accumulation in KTRG-B49. The polyamine profiles were assessed with a focus on agmatine, putrescine, cadaverine, and spermidine. As shown in Fig. 3a, the contents of agmatine and spermidine remained almost unchanged during the freezing treatment when compared with those of the CK without freezing stress group. However, freezing treatment significantly increased the levels of putrescine, and its contents were especially high (25.34–32.27 nmol g-1 FW) compared with those of the CK group (24.98 nmol g-1 FW). Furthermore, the cadaverine levels were also significantly different from those of the CK group in the 12 h recovery stage after freezing treatment, although the contents were relatively low. Therefore, these specifically increased metabolites upon freezing treatment, especially putrescine, may be related to the freezing tolerance of KTRG-B49.

### Gene correlation network analysis

First, the expression patterns of 3886 DEGs (FPKM ≥1) obtained from transcriptome sequencing were analyzed by WGCNA, and they were divided into three modules according to the similarity of their expression patterns (Fig. 3b). The numbers of unique genes in each module were 2378, 571, and 937 for the black, green, and turquoise colors, respectively. The gene expression profile of the entire module was obtained (Fig. S1). Then, the correlations between the modules and the traits were analyzed, and the data were shown in Fig. 3c. One module of interest was the turquoise module; the correlation coefficient of that module with putrescine was the highest (0.78, *P*-value = 0.00003), indicating that the module ‘Turquoise’ was closely related to putrescine accumulation in turnip. To further identify ‘Turquoise’ modular features, we used KEGG pathway analysis for deep analysis (Fig. S6). Six genes were included in the METurqoise module and were found to be involved in the ‘arginine and proline metabolism’ pathway (ko0330, M00133). Among these genes, *BrrADC2.2* (GenBank number: MN630676) was found to participate in polyamine synthesis, and the increase in *BrrADC2.2* expression was consistent with the accumulation of putrescine under freezing treatment. Taken together, these results suggested that *BrrADC2.2* may play a key role in polyamine synthesis under freezing stress in turnip, and the upregulation of *BrrADC2.2* expression and the increase in putrescine could be essential for freezing tolerance of turnip.

### Identification of upstream regulatory transcription factors of *BrrADC2.2*

**Fig. 4:**
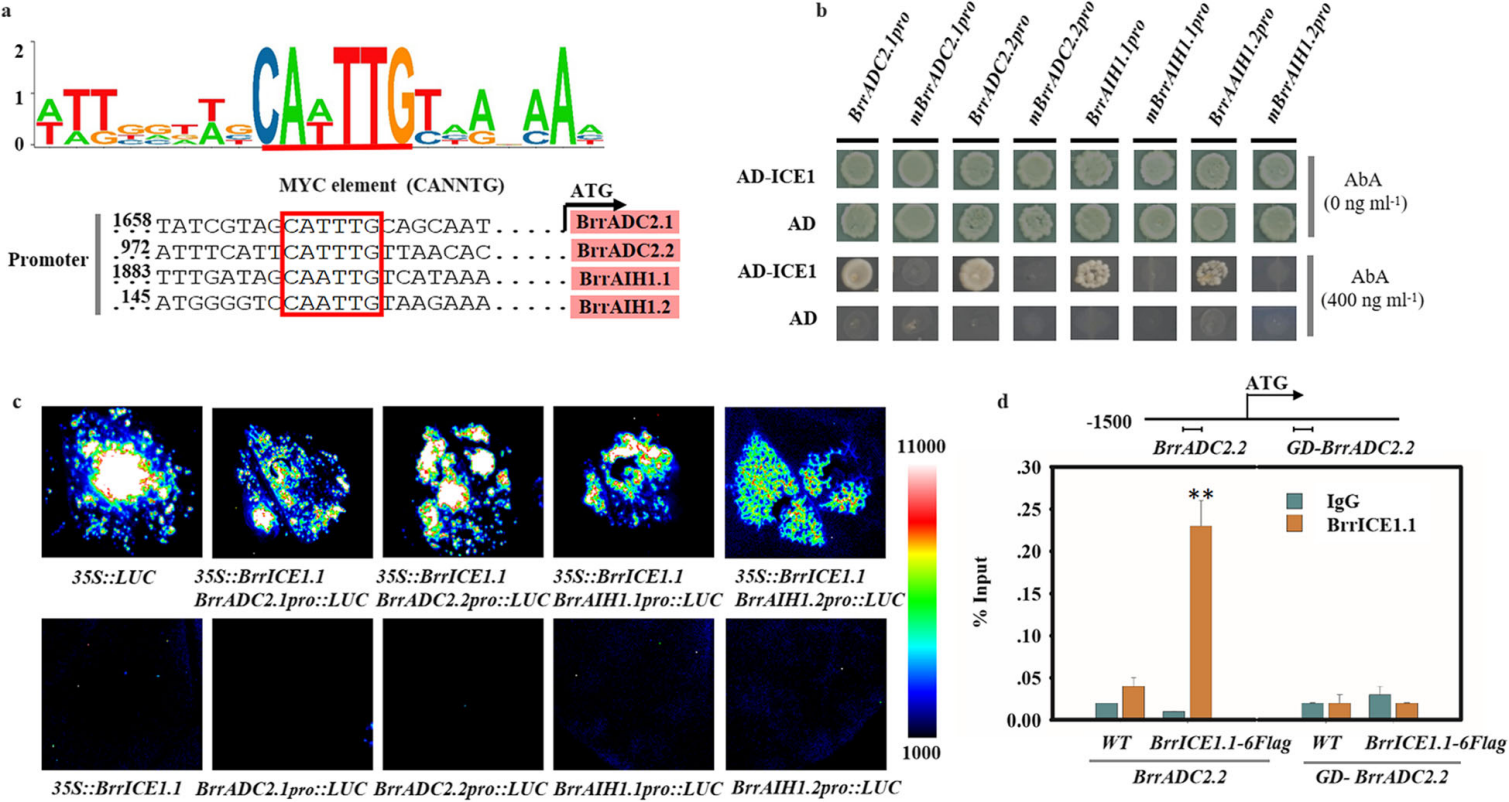
Binding motif analysis in the target regions of turnip BrrICE1.1 and interaction analysis of BrrICE1.1 with the promoter of the differentially expressed genes in the polyamine pathway in vivo and in vitro. **a** The potential MYC-binding site (CANNTG) of BrrICE1.1. The binding sequences of the BrrICE1.1 with *BrrAIH1.1*, *BrrAIH1.2, BrrADC2.1*, and *BrrADC2.2* are shown in red box. **b** Yeast one-hybrid assays showed that the MYC element mediates BrrICE1.1 binding to the *BrrAIH1.1*, *BrrAIH1.2*, *BrrADC2.1*, and *BrrADC2.2* promoters, and the *BrrAIH1.1*, *BrrAIH1.2*, *BrrADC2.1*, and *BrrADC2.2* promoters were mutated (deleted MYC element) to abolish the MYC element alone. The experiments were repeated three times with the same results. **c** BrrICE1.1 activated the activity of *BrrAIH1.1*, *BrrAIH1.2*, *BrrADC2.1*, and *BrrADC2.2* in vivo. *N. benthamiana* leaves. Representative images of *N. benthamiana* leaves 72 h after infiltration are shown. **d** ChIP experiment using *BrrICE1.1-6flag* transgenic hair root. The structure of the *BrrADC2.2* gene promoter. The primer sequence regions used for ChIP assays are marked with a horizontal line to the left of the TSS. The control primer sequence (GD) was on the left side of TSS. ChIP-qPCR showing binding of BrrICE1.1 to *BrrADC2.2* promoters in vivo. WT and *BrrADC2.2-GD* were used as negative controls. The data are the mean of three replicates ± SD, and the asterisks indicate significant differences compared with IgG (*P < 0.05, **P < 0.01, Student’s *t* test)

We performed yeast one-hybrid assays to screen the transcription factors interacting with *BrrADC2.2* promoter as bait and the turnip cDNA library as prey, and positive colonies were partially sequenced and identified by BLAST analysis. Finally, we identified a cDNA of a *B. rapa* ICE1-like transcription factor and named it *BrrICE1.1* (GenBank number: MN630673) in our turnip. BrrICE1.1 has the entire set of signature motifs required for defining a typical bHLH transcription factor (Fig. S7). In addition, ICE1 was reported to be able to bind specifically to the MYC recognition sequence (CANNTG). Thus, we further analyzed the promoter region of *BrrADC2.2* using the PlantCARE database (http://bioinformatics.psb.ugent.be/webtools/plantcare/html/). As expected, there was a potential MYC recognition element (CATTTG) (958–963) within the 1-kb region upstream of the ATG (Fig. 4a, Table S5). Therefore, we speculated that BrrICE1.1 combined with the CATTTG element in the promoter region of *BrrADC2.2* plays a role in freezing tolerance. Moreover, we analyzed the promoter sequence of other DEGs in the polyamine pathway, and an additional six DEGs (*BrrADC1*, GenBank number: MN630674; *BrrADC2.1*, GenBank number: MN630675; *BrrAIH1.1*, GenBank number: MN630677; *BrrAIH1.2*, GenBank number: MN630678; *BrrSAMDC1.1*, GenBank number: MN630679; *BrrSPD2.1*, GenBank number: MN630681) were found to contain the MYC recognition element (Table S5).

### BrrICE1.1 bound to the promoters of polyamine pathway DEGs in vitro and in vivo

Possible interactions of BrrICE1.1 with the MYC elements of the above gene promoters were tested using a yeast one-hybrid system. We found that BrrICE1.1 could activate *BrrADC2.1pro::pAbAi*, *BrrADC2.2pro::pAbAi*, *BrrAIH1.1pro::pAbAi*, and *BrrAIH1.2pro::pAbAi*, but not their mutant, suggesting that the MYC elements, CATTTG sites for *BrrADC2.1* and *BrrADC2.2*, and CAATTG sites for the *BrrAIH1.1* and *BrrAIH1.2* promoters were necessary and required for *BrrICE1.1* binding in yeast (Fig. 4b).

Next, using the well-established transient expression assay of *N. benthamiana* leaves, we further verified the transcriptional activation activity of BrrICE1 with above four genes (Fig. 4c). Coexpression of *35S::BrrICE1.1* with *BrrADC2.1pro::LUC, BrrADC2.2pro::LUC, BrrAIH1.1pro::LUC,* and *BrrAIH1.2pro::LUC* could detect *LUC* activity, indicating that BrrICE1.1 activated their expression, respectively. Furthermore, ChIP experiments using *BrrICE1.1-6Flag* transgenic hair roots and an anti-Flag antibody were employed to confirm the binding of BrrICE1.1 to these four gene promoters in vivo. qPCR revealed that only the BrrICE1.1-6Flag protein could immunoprecipitate the *BrrADC2.2* promoter region containing an element of CATTTG (compared to the IgG and WT) (Fig. 4d). However, *BrrADC2.1*, *BrrADC2.2*, *BrrAIH1.1* and *BrrAIH1.2* were not detected. Together, these data suggested that BrrICE1.1 could directly bind to the promoter of *BrrADC2.2* and that the CATTTG elements presented in the *BrrADC2.2* promoter were the binding sites for BrrICE1.1.

### Transgenic and RNAi hair roots showed altered levels of *BrrADC2.2* transcripts and polyamines

**Fig. 5:**
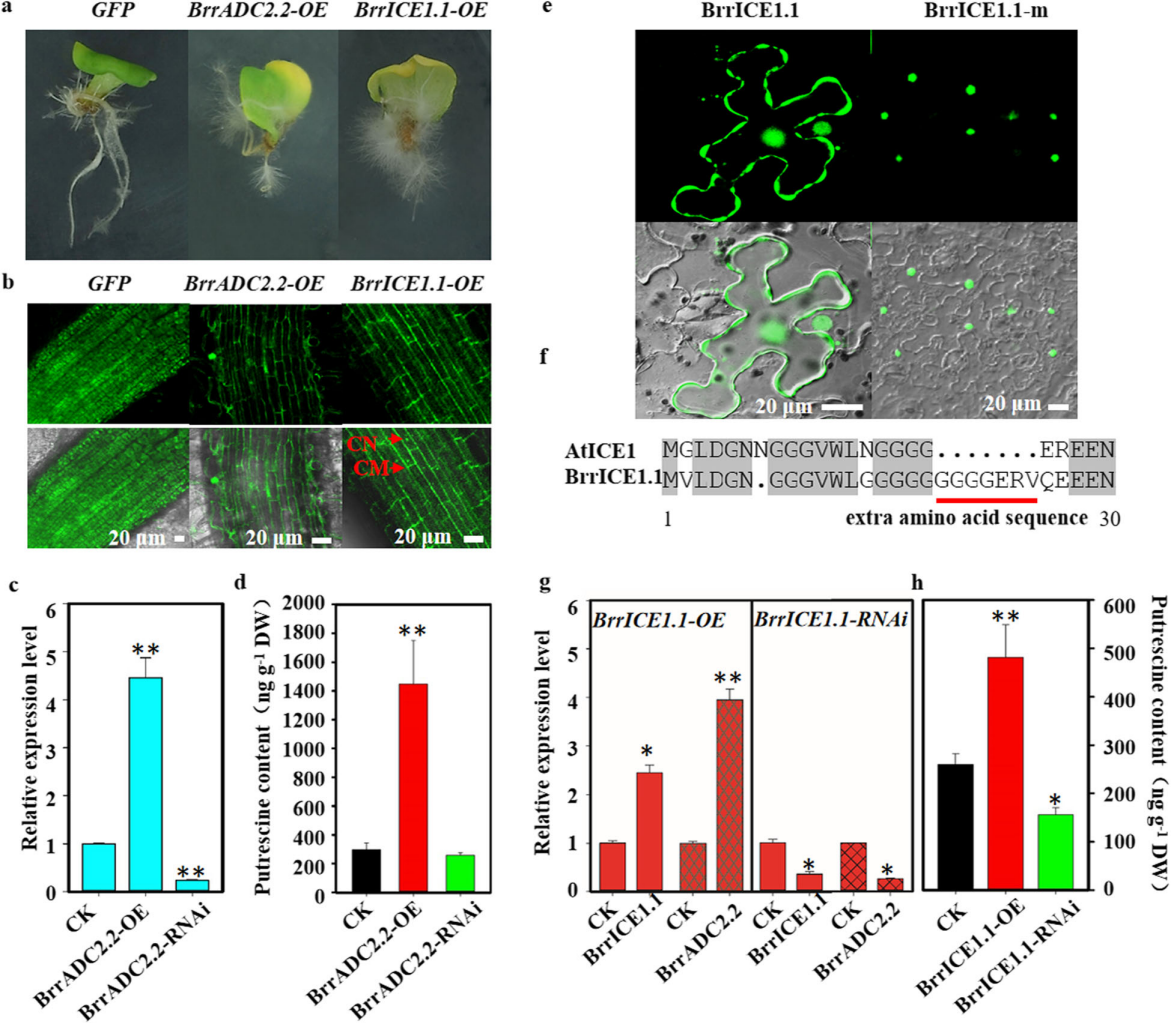
Detection of expression and putrescine contents in transgenic and RNAi hairy roots. **a** The phenotype of the hair root (GFP, control; *BrrADC2.2-OE*, overexpression of *BrrADC2.2* in hair root; *BrrICE1.1-OE*, overexpression of *BrrICE1.1* in hair root). **b** Laser confocal detection of overexpressed *GFP*, *BrrADC2.2-OE* and *BrrICE1.1-OE* in the hairy roots. CN: cell nucleus, CM: cell membrane. Bar = 20 μm. **c d** The expression levels of *BrrADC2.2* and putrescine contents in *BrrADC2.2-OE* transgenic and *BrrADC2.2-RNAi* hairy roots compared with those of the CK. **e** Laser confocal detection of overexpressed *BrrICE1.1* and its amino acid sequence mutant (BrrICE1.1-m) protein localization in *N. benthamiana* leaves. Bar = 20 μm. **f** The structure of the AtICE1 and BrrICE1.1 protein at the N-terminus (1–30). The red line represents a single peptide in the BrrICE1.1 protein. **g** The expression levels of *BrrICE1.1* and *BrrADC2.2* in *BrrICE1.1-OE* transgenic hair roots (left) and *BrrICE1.1* and *BrrADC2.2* in *BrrICE1.1-RNAi* transgenic hair roots (right). **h** Putrescine contents in *BrrICE1.1-OE* transgenic and *BrrICE1.1-RNAi* hairy roots. In **c**, **d**, **g** and **h**, the data are the mean of three replicates ± SD, and the asterisks indicate significant differences compared with the CK (*P < 0.05, **P < 0.01, Student’s *t* test)

The effect of the *BrrADC2.2* gene on putrescine synthesis was further verified by using *A. rhizogenes*-mediated infection of the cotyledons of turnip to obtain *BrrADC2.2* transgenic and RNAi hairy roots (Fig. 5a). Fluorescence of the hairy roots overexpressing *BrrADC2.2-OE* was obtained by confocal laser microscopy (Fig. 5b). Moreover, we tested the expression levels of *BrrADC2.2* in *BrrADC2.2-OE* and *BrrADC2.2-RNAi* using qRT-PCR analysis (Fig. 5c). The expression of *BrrADC2.2* was significantly up- and down-regulated in *BrrADC2.2-OE* and *BrrADC2.2-RNAi* hair roots, respectively, compared with the control, indicating that the *BrrADC2.2* gene was successfully expressed in the roots. Additionally, the putrescine content of transgenic *BrrADC2.2-OE* measured by LC-MS was significantly higher than that of the control. As expected, *BrrADC2.2-RNAi* reduced the putrescine content (Fig. 5d). Both the *BrrADC2.2* expression levels and putrescine contents indicated that *BrrADC2.2* displayed a crucial role in putrescine synthesis.

### *BrrICE1.1* directly regulated *BrrADC2.2* in putrescine synthesis

To identify the role of *BrrICE1.1* in putrescine synthesis, we obtained transgenic and RNAi hairy roots (Fig. 5a). The BrrICE1.1-GFP fusion protein emitted a green fluorescent signal in the membranes and nuclei of the hair roots (Fig. 5b). Interestingly, cell membrane localization of BrrICE1.1 was present in turnip but absent in *Arabidopsis* (Fig. 5e, left). To explore the membrane localization of turnip BrrICE1.1, we analyzed the amino acid sequence characteristics of BrrICE1.1 and AtICE1 (Fig. 5f). We found that the C-terminus was conserved, while at the N-terminus, BrrICE1.1 had an extra amino acid sequence (GGGGERV). We speculated that this amino acid sequence affected the location of BrrICE1.1. Hence, we deleted it to test the location of BrrICE1.1 and we were surprised to find that its membrane localization disappeared and only nuclear localization was observed in *N. benthamiana* leaves (Fig. 5e, right). Therefore, this extra amino acid sequence was indeed the reason for the membrane localization of BrrICE1.1.

Efforts were further made to compare the expression levels and putrescine contents among transgenic, RNAi and WT hairy roots. The expression level of *BrrICE1.1* was slightly upregulated and *BrrADC2.2* was significantly upregulated in transgenic *BrrICE1.1-OE* hairy roots compared to WT roots, while the expression levels in *BrrICE1.1-RNAi* hairy roots were lower than those in the WT (Fig. 5g). With respect to putrescine contents, LC-MS measurement showed that transgenic lines increased the levels of putrescine, in contrast to a reduction in the RNAi hairy roots (Fig. 5h). The expression level analysis together with the putrescine content analysis presented here may indicate that *BrrICE1.1* positively regulates the expression of *BrrADC2.2* in freezing stress of turnip.

### Effect of exogenous application of putrescine and its inhibitor on the freezing tolerance of turnip

**Fig. 6:**
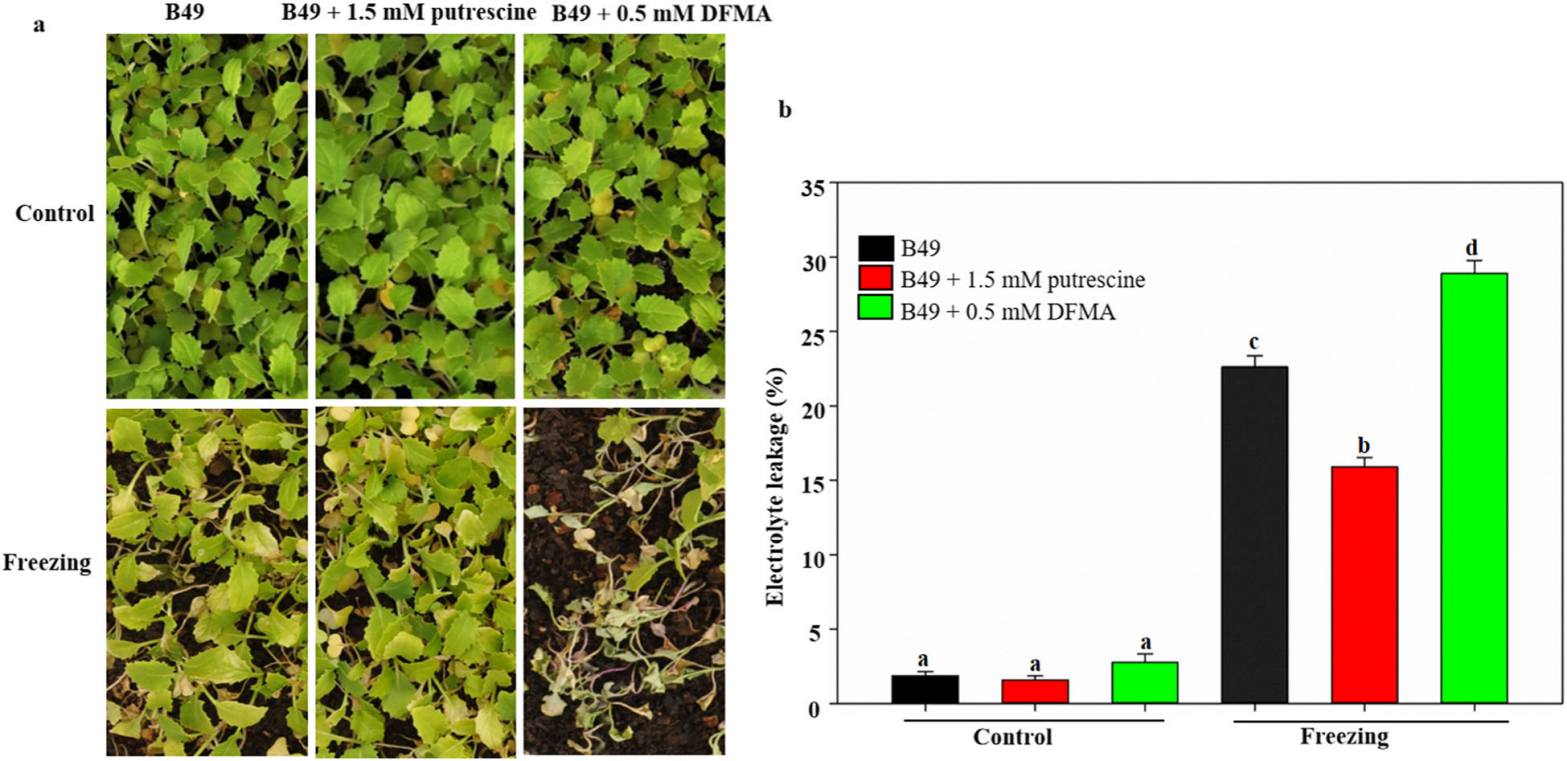
Effects of exogenous putrescine and its inhibitor on freezing tolerance of KTRG-B49. **a** Representative plants of KTRG-B49 with and without 1.5 mM putrescine and 0.5 mM DFMA before (Control) and after freezing treatment (Freezing), respectively. **b** Electrolyte leakage of leaves from control and freezing treated plants. Values are means of 5–8 biological replicates. Bars indicate SD. Different symbols indicate significant differences between treatments (*P* < 0.05) according to Tukey’s test

To confirm the role of putrescine in freezing tolerance in turnip, exogenous putrescine and inhibitor (DFMA) were applied to the KTRG-B49 under freezing conditions, respectively (Fig. 6). There were no obvious changes in plant morphology between putrescine-treated, inhibitor-treated and control plants before application of the freezing treatments. When submitted to freezing treatment, the putrescine-treated plants suffered significant less injury compared with the control, whereas the inhibitor-treated plants suffered the most severely damage (Fig. 6a). To further confirm the results obtained above, electrolyte leakage was used to measure the plants after freezing treatment. Consistently, electrolyte leakage of putrescine-treated leaves exhibited a significantly lower electrolyte leakage after freezing treatment (Fig. 6b). These results suggested that putrescine could play an important role in turnip freezing tolerance.

### The CBF signaling pathway was enhanced by freezing stress in KTRG-B49

**Fig. 7:**
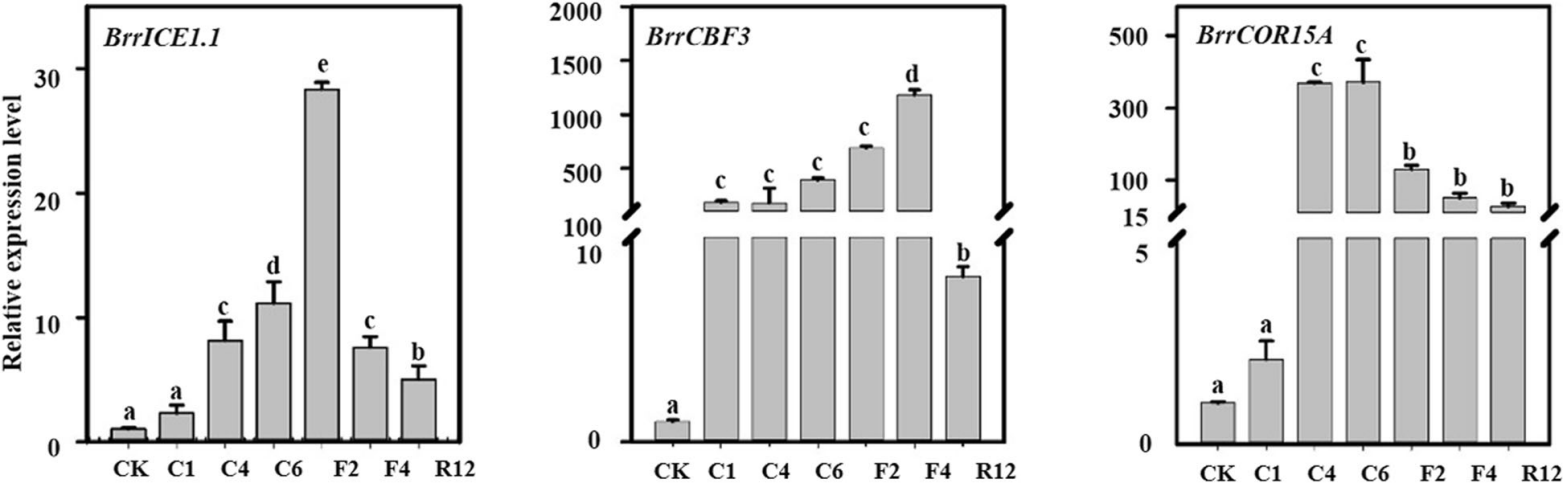
Expression of the *BrrICE1.1*, *BrrCBF3* and *BrrCOR15A* genes upon freezing treatment with three biological and technical replicates. Different symbols indicate significant differences between treatments (*P* < 0.05) according to Tukey’s test

**Fig. 8:**
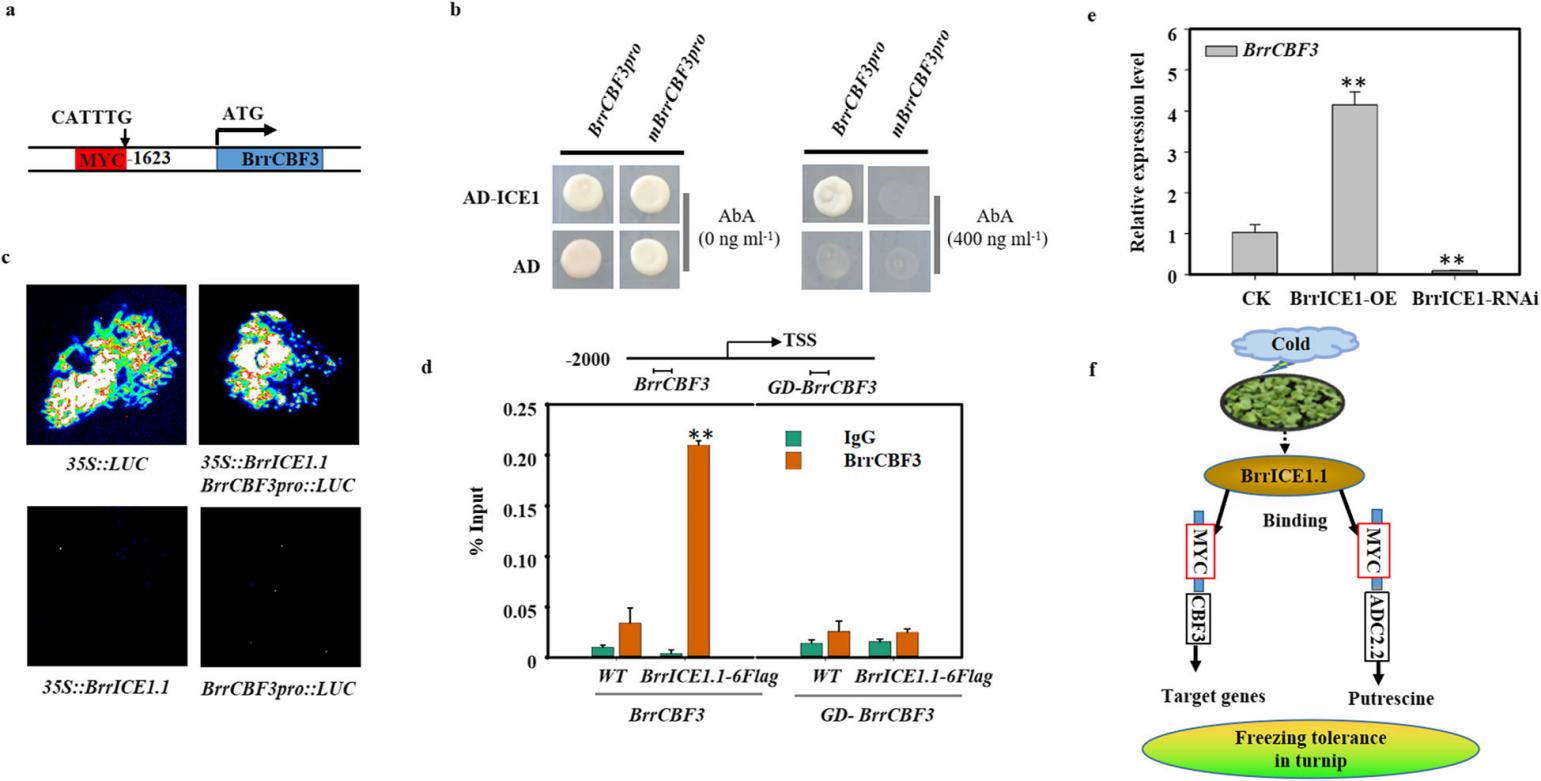
BrrICE1.1 bound to the *BrrCBF3* promoter. **a** The potential binding site of BrrICE1.1 is the MYC element (CATTTG) in the *BrrCBF3* promoter. **b** Yeast one-hybrid assays showed that the MYC element mediates BrrICE1.1 binding to the *BrrCBF3* promoter and that the *BrrCBF3* promoters were mutated (deleted MYC element) to abolish the MYC element alone. The experiments were repeated three times with the same results. **c** BrrICE1.1 activated the binding activity of BrrCBF3 in vivo; *N. benthamiana* leaves were transformed with the positive control (*35S*::*LUC*) and negative control (*35S::BrrICE1.1*, *BrrCBF3pro::LUC*), and the interaction was detected. Representative images of *N. benthamiana* leaves 72 h after infiltration are shown. **d** ChIP experiment using *BrrICE1.1-6flag* transgenic hair root. The structure of the *BrrCBF3* gene promoter. The primer sequence regions used for ChIP assays are marked with a horizontal line to the left of the TSS. The control primer sequence (GD) is on the left side of the TSS. ChIP-qPCR showing binding of BrrICE1.1 to the *BrrCBF3* promoters in vivo. WT and *BrrCBF3-GD* were used as negative controls. **e** The expression of *BrrCBF3* in *BrrICE1.1-OE* and *BrrICE1.1-RNAi* transgenic hair roots. **f** BrrICE1.1 regulatory network under freezing stress in turnip. In **d** and **e**, the data are the mean of three replicates ± SD, and the asterisks indicate significant differences compared with the control (*P < 0.05, **P < 0.01, Student’s *t* test)

CBF represents a well-documented pathway involved in plant cold responses. To clarify the possible signal transduction, we monitored the expression levels of *BrrICE1.1*, *BrrCBF3* and the downstream *BrrCOR15A* genes in turnip under freezing stress. We observed an elevation of all three genes, although significance was not observed in all periods of the freezing treatment (Fig. 7). Next, we asked whether BrrICE1.1 regulated *BrrCBF3* using in vivo and in vitro methods. We first analyzed the promoter region of *BrrCBF3* and found that it contained the MYC recognition element (CATTTG) in a 1623 region (Fig. 8a), and thus, we confirmed that BrrICE1.1 could bind to *BrrCBF3*. Indeed, Y1H assays revealed that BrrICE1.1 could activate *BrrCBF3*_*pro*_*::pAbAi*, suggesting that the BrrICE1.1 binding sites may be CATTTG. The activation of BrrICE1.1 on the reporters was completely abolished when CATTTG was deleted (*mBrrCBF3*_*pro*_), suggesting that the CATTTG element in the *BrrCBF3* promoter was necessary and required for BrrICE1.1 binding in yeast (Fig. 8b). We thus performed LUC assays to determine the effect of BrrICE1.1 on the expression of a reporter containing the *BrrCBF3* promoter fused with the LUC reporter gene. We found that coexpression of *35S::BrrICE1.1* with the *BrrCBF3pro::LUC* reporter led to an obvious increase in LUC activity, indicating that BrrICE1.1 activated the expression of *BrrCBF3* (Fig. 8c). To confirm this effect, we used ChIP-qPCR analysis. An IgG antibody and WT were used as controls, and two different sets of primers, *CBF3* promoter primers containing CATTTG elements (*BrrCBF3*) and *CBF3* gene background primers (*BrrCBF3-GD*), were used to test the BrrICE1.1-6Flag immunoprecipitated complexes. Notably, *BrrCBF3* promoter primer enrichment was significantly increased in BrrICE1.1-6Flag immunoprecipitated complexes (Fig. 8d). This finding implied that BrrICE1.1 directly regulated *BrrCBF3*. To examine the function of *BrrCBF3* and *BrrICE1.1*, we generated *BrrICE1.*1 overexpression and RNAi hair roots. Overexpression of *BrrICE1.1* led to enhanced induction of the *BrrCBF3* gene, whereas the expression level of *BrrCBF3* was downregulated in the *BrrICE1.1-RNAi* hair roots (Fig. 8e). These investigations suggested that the *BrrICE1.1* signal pathway could be associated with CBF-mediated freezing tolerance in turnip.

## Discussion

Low temperature restricts the growth and geographical distribution of plants, and thus, major crop losses caused by low temperature injury are observed every year worldwide [27]. In the agricultural area of the Qinghai-Tibet Plateau, the average temperature of the crop growing season is very low, and extreme temperature has become one of the limiting factors of crop yield. Therefore, it is necessary to reveal the metabolism and molecular mechanisms of crops under freezing stress, which will help cultivate freezing-tolerant crop varieties, thereby reducing production losses. Tibetan turnip is suitable for cultivation in the alpine region due to its freezing resistance; this plant showed strong freezing tolerance and good adaptability to the climate in Tibet [26]. In turnip, the pathways responding to freezing stress have not been systematically investigated. Analysis of the adaptability of turnip to a low-temperature environment will help elucidate the molecular mechanism of freezing tolerance of turnip and could also provide suggestions for breeding turnip in Tibet. In our study, we first showed that turnip KTRG-B49 could resist freezing tolerance and to some extent elucidated the characteristics of freezing resistance of turnip in Tibet (Fig. 1).

### The increase in *BrrADC2.2* expression is consistent with the accumulation of putrescine under freezing treatment

Upon exposure to cold stress, the metabolic and physiological status of plants is altered, which is accompanied by changes in the expression of thousands of genes [18]. For example, polyamine has been suggested to be a crucial factor in the response of plants to low temperature [12, 24]. In the current study, expression profile analysis of differentially expressed genes (DEGs), which was similar to PCA analysis with a transcriptome background, showed enrichment in the polyamine pathway (Fig. 2a, b, c, Fig. S4). Furthermore, polyamines, particularly putrescine (Put), have been proposed to play an important role in the ability of plants to cope with cold stress [6]. In our study, free putrescine continued to accumulate rapidly and specifically under low-temperature stress, reaching a high level (Fig. 3a). In addition, the content of polyamines in plants can be used as an important index to measure their resistance to stress, as polyamine-rich plants usually show high resistance to stress [9, 13, 28, 29]. However, no noteworthy changes were found in the agmatine and spermidine contents. Similar results indicated that putrescine’s downstream product spermidine did not increase with putrescine content [6], and another study reported that the putrescine content of *A. thaliana* and *Thellungiella halophila* was improved, but its precursors showed no significant variation under cold treatment [30]. It was thus suggested in our study that the specific increase in the putrescine content may be closely related to the stress signal in response to low-temperature stress and may slow the damage due to low temperatures and enhance the freezing resistance in turnip.

The polyamine pathway is complex and involves many key enzymes [1, 6], including putrescine, which was synthesized in one step by ODC or in three steps by ADC, AIH and CPA [2, 3]. Our WGCNA revealed that the expression of *BrrADC2.2* showed accumulation along with the putrescine contents, indicating that *BrrADC2.2* was involved in the synthesis of endogenous putrescine under freezing treatment in turnip (Fig. 3b, c). In *Arabidopsis*, overexpression of the *ADC2* gene increased the freezing tolerance of transgenic plants [31, 32] and *adc2* mutants could restore freezing tolerance to wild-type levels by exogenous putrescine application [14]. Interestingly, induction of *ADC2* was also associated with osmotic stress, wounding, light, sucrose, flower development, seed germination, and salinity [31–35]. Thus, *BrrADC2.2*-catalyzed putrescine synthesis may promote the freezing tolerance of turnip, and these results provide useful information for further research on the molecular mechanism by which *BrrADC2.2* participates in freezing tolerance in turnip.

### BrrICE1.1 directly binding to the *BrrADC2.2* promoter regulates the synthesis of putrescine

At present, there are few studies on the regulatory relationship between the *ADC* and other genes. Prior research only pointed out that the *ADC1*-associated putrescine pathway plays an important role in the cold-acclimated freezing tolerance of potatoes, most likely by enhancing the expression of *CBF* genes [6]. In the current study, *BrrADC2.2* was activated by BrrICE1.1 of turnip, as shown through in vivo and in vitro assays (Fig. 4). And overexpression of *BrrICE1.1* increased the levels of putrescine (Fig. 5). Meanwhile, overexpression of *PtrICE1* could increase the levels of spermine and spermine in transgenic lines based on the ADC interaction with PtrICE1 by a yeast two-hybrid assay in *P. trifoliata*. However, it was not clear which polyamine pathway gene acts on PtrICE1 to regulate polyamine level [24]. In the process of plants responding to cold, ICE1 plays a critical role in response to environmental changes by positively regulating genes through binding specifically to the MYC element in the promoter region which is considered as a classical mode of action on ICE1 [20, 36]. Our results of *BrrADC2.2* and *BrrICE1.1* transgenic and RNAi roots showed that the increase of putrescine content by overexpression of *BrrICE1.1* was mainly due to the binding of BrrICE1.1 to the MYC element of *BrrADC2.2* promoter region in turnip (Fig. 5). And, it remains to be investigated whether greater activation of *ADC* genes in the transgenic lines results from cold-induced post-transcriptional modification of BrrICE1.1, such as sumoylation and ubiquitination. Thus, our data demonstrated that BrrICE1.1 played a positive role in putrescine synthesis, likely in response to freezing tolerance through binding to *the BrrADC2.2* promoter. Moreover, foliar applied putrescine due to their ability to act as growth regulator is able to modulate the plant metabolism and the production of metabolites involved in stress tolerance [6]. In our research, both the exogenous application of putrescine and its inhibitor further confirmed the function of putrescine under freezing tolerance in turnip (Fig. 6).

### The reason of the cell membrane localization of the BrrICE1.1 protein

Notably, the GFP-BrrICE1.1 fusion protein was located in the cell membrane and nucleus in our study (Fig. 5e, f), in contrast to the nuclear localization of GFP-AtICE1 in *Arabidopsis* [20]. By deleting the N-terminal extra single peptide (GGGGERV) of the BrrICE1.1 protein compared with AtICE1, the membrane localization of the BrrICE1.1 protein disappeared, suggesting that this single peptide affected the location of the BrrICE1.1 protein. In turnip, this transcription factor with nuclear location and cell membrane location was found for the first time. The biological significance or role of more accurate cell membrane location needs further study. In *Arabidopsis*, this membrane-associated transcription factor has also been studied (such as *NAC089*). The existence of membrane localization of NAC089 protein ensures that *Arabidopsis* can make rapid transcription response to external stimulation, and can transfer from endoplasmic reticulum to cell approved site under stress [37, 38]. The disparity of cell localization between BrrICE1.1 and AtICE1 might be presumably ascribed to the inherent difference in plant species, which is an important issue for future research. These findings provide new knowledge of the function and underlying mechanism of ICE1 and expand our understanding of the complex cold signaling network.

### BrrICE1.1-BrrCBF3 pathway is involved in the freezing stress of the turnip

Here, we could not eliminate the contribution of other signaling pathways besides the putrescine pathway to the freezing tolerance of turnip, so we attempted to link it with known freezing stress-related signaling pathways. The CBF regulatory pathway is known to be one of the most important pathways and plays a key role in cold signal transduction in many species [20, 39–41]. In current study, freezing stress led to enhanced induction of CBF3 and the downstream COR genes (Fig. 7). Moreover, BrrICE1.1 was shown to bind to the promoters of *BrrCBF3* at CATTTG sites (Fig. 8a, b, c, d). This finding was similar in *Arabidopsis* because the ICE1 could bind directly to the canonical *MYC cis*-elements (CANNTG) in the *CBF3* promoter [20]. Additionally, changes in the *BrrCBF3* expression patterns were consistent with those in the hair roots with overexpression and silencing of *BrrICE1.1* (Fig. 8e), implying that *BrrICE1.1* played a positive role in *BrrCBF3*-mediated freezing signaling in turnip.

## Conclusions

Based on the results obtained in this study that transcriptomics and targeted metabolomics analyses provided new insights into freezing tolerance in Tibetan turnip, a working model of the BrrICE1.1 regulatory network involved in the turnip freezing process was proposed (Fig. 8f). When turnip is subjected to freezing stress, BrrICE1.1 can not only regulate the downstream target gene through ICE1-CBF pathway to participate in turnip freezing damage, but also combine with *BrrADC*2.2 promoter to regulate the synthesis of putrescine, so as to participate in turnip freezing regulation. The above-mentioned illustration suggested that modulation of cellular polyamine content could be regarded as a convenient and effective strategy to enhance freezing tolerance.

## Methods

### Plant cultivation, freezing treatment and electrolyte leakage measurement

Seeds of *Brassica rapa var. rapa* (KTRG-B49) were obtained from the Germplasm Bank of Wild Species, Kunming Institute of Botany, Chinese Academy of Sciences, Kunming, China, which were collected from Basu County, Changdu District, Tibet, China (N30°03′25.76″, E96°55′37.70″). The harvested seeds were sown in a seedling-raising plate with appropriate watering under controlled greenhouse conditions (28 °C day/25 °C night, 200 mmol photons m^− 2^ s^− 1^ light intensity, relative humidity of 75–80%) at Kunming Institute of Botany. For freezing treatment, 2-week-old plants (approximately 100 seedlings in each experiment) were assayed as described [42]. Briefly, the plants were incubated at 4 °C for 6 h. Then, the temperature was successively decreased by 2 °C h^− 1^ until reaching − 2 °C and was held at − 2 °C for 2 h and − 4 °C for 1 h in a programmed incubator. Next, the plants were kept overnight at 4 °C for 12 h and then returned to the greenhouse conditions for recovery, followed by an assessment of the survival rate. In the meanwhile, untreated plants used as control, and other conditions were unchanged. Leaf-samples (randomization approach) were collected from the plants after freezing treatment for 0 h (CK), 4 °C for 1 h (C1), 3 h (C4), 6 h (C6), − 2 °C for 2 h (F2), − 4 °C for 1 h (F4) and recovery for 12 h (R12), then immediately frozen in liquid nitrogen and then stored at - 80 °C. Collection was performed from more than 3 plants at each sampling time, and each sample collection was repeated 3 times to obtain biological replicates (the three biological replicates included three separate set of freezing stress treatment experiments). There were 21 samples in total used for RNA-seq and metabolite analysis. Also, the electrolyte leakage was used to evaluate freezing tolerance at each sampling time following a previously described method [43].

### Illumina RNA-sequencing and data analysis

Total RNA was isolated from 21 leaf-samples and messenger RNA (mRNA) was purified and used to generate short fragments via fragmentation buffer to synthesize the first-strand cDNA and the second cDNA. The fragments of double-stranded cDNA were retrieved and connected with sequencing adapters. Then, the suitable fragments were isolated by Agencourt AMPure XP beads (Beckman Coulter, Inc.) and enriched by PCR amplification. The constructed cDNA libraries were paired-end 100-bp sequenced using an Illumina HiSeq 2000 system. The RNA-seq raw sequence data reported in this paper have been deposited in the NCBI (PRJNA667167) that are publicly accessible at https://www.ncbi.nlm.nih.gov/bioproject/PRJNA667167.

Clean reads were obtained by removing low quality reads using perl script, such as only adapters and unknown (‘N’ > 5%) or low-quality bases (scores < 20). The clean reads were mapped to turnip reference genome [44] using TopHat 2.0.8 program [45]. Differential expression analysis was performed using the DESeq R package (1.10.1) [46]. And FPKM (fragments per kilobase of exon per million mapped reads) was used to estimate abundance differences by Cufflinks normalization (http://cufflinks.cbcb.umd.edu/. The false discovery rate (FDR) ≤ 0.001 and fold change ≥4 were used as the threshold to judge the significance of gene expression differences (DEG). Sequences were compared against various protein databases using BLASTx, including the NCBI non-redundant protein (Nr) database, Swiss-Prot by a cutoff e-value of 10^− 5^. Gene ontology (GO) terms, Clusters of Orthologous Group (COG) database and KEGG pathways were used to annotate the genes.

### RNA extraction, quantitative real-time PCR (qRT-PCR)

Total RNA extraction, cDNA synthesis and qRT-PCR analysis were performed as described previously [25]. The primers used for qRT-PCR are listed in Table S1. Statistical analysis was performed using the software IBM SPSS Statistics 20.0.

### Metabolite extraction and derivatization

An aliquot of each individual sample was precisely weighed and transferred to a centrifugal tube. The samples were extracted with 500 μL acetonitrile: methanol: water = 2: 2: 1 (precooled at − 20 °C), and then homogenized at 45 Hz for 4 min with vortex for 30 s, sonicated treatment in an ice-water bath for 5 min. The above procedure was repeated 3 times, then incubated at − 20 °C for 1 h and centrifuged to obtained supernatant. Then, 200 μL of supernatant was used for LC-MS analysis. For UHPLC-MS/MS analysis, a 100 μL aliquot of the clear supernatant (or standard solution) was further transferred to a centrifugal tube and then mixed with 50 μL of 20 mg/mL dansyl chloride in acetone and 50 μL of 0.1 mol/L sodium carbonate after 60 min incubation at 40 °C in the dark. Dansyl derivatives were added to 50 μL of 1% formic acid in water, and the samples were vortexed for 30 s and centrifuged at 12000 rpm and 4 °C for 15 min. An 80 μL aliquot of the clear supernatant was transferred to an autosampler vial for UHPLC-MS/MS analysis.

### Weighted gene coexpression network analysis (WGCNA)

WGCNA was performed using the R package (version 3.4.1) to construct a gene coexpression network. The genes with FPKM values > 1 used for the network were based on the above RNA-seq data from 7 samples of different freezing treatments, using each biological and technical replicate as an individual dataset (a total of 21 samples). A topological overlap matrix was constructed with a threshold power of 10 (Fig. S1), and the TOM similarity algorithm was used to transform the adjacency matrix into a topological overlap matrix to reduce noise and false correlation. A dynamic tree cut procedure (mergeCutHeight = 0.25, minModuleSize = 30) was performed to identify similar modules in a hierarchal clustering tree. Different branches of the cluster tree represented different gene modules. If identified as the same module, then the relationship between these genes within the module is relatively close. The weighted average of the gene expression profile of each module was defined as the module eigengene (ME). The heatmap of correlation between MEs and stress stimulations showed the relationships between modules and given traits. The higher correlation between the trait and the module, it was likely that the trait was related to the gene function of the module.

### Yeast one-hybrid (Y1H) screening

The Y1H experiment was conducted using the Matchmaker™ Gold Yeast One-Hybrid Library Screening System (Clontech) according to the manufacturer. The promoter region (2000 bp upstream from the initiation codon) of *BrrADC2.2* was cloned into the *pAbAi* vector as bait and transformed into Y1HGold. The antibiotic resistance was tested on SD medium lacking Ura in the presence of aureobasidin A (SD/Ura + AbA). Then, a cDNA pool of turnip and a SmaI-linearized *pGADT7-RecAD* cloning vector were cotransformed into the Y1HGold strain that had been created. Positive clones were screened by yeast colony PCR, and the PCR products were then analyzed by sequencing.

For the yeast one-hybrid validation assays, the promoters of *BrrADC1*, *BrrADC2.1*, *BrrADC2.2*, *BrrAIH1.1*, *BrrAIH1.2, BrrSAMDC1.1*, *BrrSPD2.1* and *BrrCBF3* were also separately cloned into the *pAbAi* vector, and the CDS of *BrrICE1.1* was subcloned into the *pGADT7* vector. For generation of these promoters with mutations, site-directed mutagenesis was used to delete the CANNTG motif of these promoters using the TaKaRa MutanBEST kit. Yeast one-hybrid assays were carried out using the Frozen-EZ Yeast Transformation II Kit (Zymo Research, Irvine, CA, USA). The antibiotic resistance was tested on SD medium lacking Ura in the presence of aureobasidin A (SD/Ura + AbA). The DNA-protein interaction was selected on SD (−Ura /Leu + AbA) medium. The primers used in this study are listed in Table S1.

### Transient expression assays in *Nicotiana benthamiana* leaves

The *luciferase* gene (*LUC*) sequence was recombined into the empty vector *PRI101-AN-Flag*, and the positive control plasmid *35S::luciferase-6Flag* was obtained. The *BrrADC2.1pro::LUC, BrrADC2.2 pro::LUC, BrrAIH1.1pro::LUC, BrrAIH1.2pro::LUC* and *BrrCBF3pro::LUC* reporter constructs were generated using an approximately 2000 bp promoter sequence fused with the *LUC* reporter gene using the HindIII and SalI sites of the *pRI101-AN DNA* vector. All primers used in this experiment are listed in Table S1. A similar approach was used to generate the *35S::BrrICE1.1-6Flag* construct. The *35S::BrrICE1.1-6Flag* plasmids were coinfiltrated with the *pro::LUC* reporter gene into *Nicotiana benthamiana* leaves using the *Agrobacterium tumefaciens* EHA105 strain. Then, the infiltrated plants was captured LUC images and determined the luminescence intensity by a Tanon 5200 automatic chemiluminescence image analysis system (Tanon, Shanghai, China). At least five independent LUC quantifications were assessed, with similar results.

### *Agrobacterium rhizogenes*-mediated transformation

The CDSs of *BrrICE1.1* and *BrrADC2.2* and their reverse complementary sequences were cloned into *PRI101-AN-Flag* vectors. Then, these constructs were separately transferred into *A. rhizogenes* LBA9402 by electroporation [47]. Cotyledon infection was used to transform the above genes into turnip roots, and the gene expression levels and putrescine content in the hair roots were extracted and analyzed by qRT-PCR and LC-MS, respectively, as described above. The transgenic hair roots of *BrrICE1.1-6Flag* were also used for chromatin immunoprecipitation (ChIP) assays. The primers used in this study are listed in Table S1.

### ChIP assay

The ChIP experiment was carried out as described with minor modifications [48]. Briefly, 2 g of *BrrICE1.1-6Flag* transgenic hairy roots and empty *6Flag* vector (WT) hairy roots were crosslinked in 1% formaldehyde buffer. The fixed roots were extracted and washed to purify the nuclei. The nuclei were suspended in nuclei lysis buffer, and the chromatin solution was sonicated to shear the DNA (kept on ice, sonication time 30 s, interval time 30 s, 30 times, power 400 w, JY92–2). A sonicated mixture (SDS concentration = 0.1%) was added to ChIP dilution buffer and incubated with DYKDDDDK Tag (D6W5B) rabbit mAb (Bibds to same epitope as Sigma’s Anti-FLAG M2**®** Antibody) and negative control antibody lgG. The protein-DNA complexes were collected by incubation with equilibrated Protein A + G Magnetic beads (LOT: 2923270). Then, the bead-protein-DNA complexes were washed and protein-DNA complexes were released by incubation with ChIP elution buffer. Then, 5 M NaCl was used to reverse the crosslinking. After the immunoprecipitated DNA was purified, qPCR was conducted to measure the *BrrADC2.1/BrrADC2.2/BrrAIH1.1/BrrAIH1.2/BrrCBF3* promoter fragment levels. Gene body (GD) primers were also used as a control. The primers used are shown in Table S1.

### Subcellular localization

Subcellular localization assays were conducted in *N. benthamiana* leaves. The coding sequence (CDS) of *BrrICE1.1* was cloned and fused into the binary vector *PRI101-AN DNA* with a green fluorescent protein (GFP) and were driven by the *cauliflower mosaic virus 35S* promoter, forming a *35S::BrrICE1.1-GFP* construct. Further, we deleted the extra amino acid sequences (GGGGERV) on *BrrICE1.1-GFP* construct and sent it to Shanghai Generay Bioteh Co. Ltd. for synthesis to obtain *BrrICE1.1-m-GFP* construct. *A. tumefaciens* EHA105 carrying the construct of interest was infiltrated into *N. benthamiana* leaves. Fluorescence images were obtained as described previously [25]. The primers used are listed in Table S1.

### Application of exogenous putrescine and its inhibitor DL-α-(Difluoromethyl) arginine (DFMA)

Exogenous putrescine and its inhibitor (DFMA) were applied to the KTRG-B49 under freezing conditions, respectively, to test the effects of putrescine on turnip freezing tolerance. The experiments were previously dscribed [49]. The 2-week-old plants were sprayed with 1.5 mM putrescine and 0.5 mM DFMA once a day for 3 days, respectively, water was used in the control. The solutions were all supplemented with 0.01% (v/v) Tween 20 as a detergent. The plants were incubated at 4 °C for 6 h. Then, the temperature was successively decreased by 2 °C h^− 1^ until reaching − 2 °C and was held at − 2 °C for 2 h, − 4 °C for 1 h and − 6 °C for 2 h in a programmed incubator. Next, the plants were kept overnight at 4 °C for 12 h and then leaves were used for the measurement of lectrolyte leakage.

## Supplementary information

**Additional file 1: Figure S1.** The decision of power value. **a** The horizontal axis represents different power values. **b** The average network connectivity under different power values. **c** Network heatmap, the gene expression profile of the entire module.

## Data Availability

The data sets generated or analyzed during this study were included in this published article and its additional files. All the transcriptome data from 21 samples have been deposited in the NCBI under SRA accession number PRJNA667167 (https://www.ncbi.nlm.nih.gov/bioproject/PRJNA667167).

## Abbreviations

BrrICE1.1: Inducer of CBF expression 1
BrrCBF: C-repeat binding factor;
BrrADC2.2: Arginine decarboxylase gene
COR: Cold-regulated
ADC: Arginine decarboxylase
ODC: Orn decarboxylase
AIH: Agmatine iminohydrolase
CPA: N-carbamoylputrescine amidohydrolase
SPDS: Spermidine synthase
SPMS: spermine synthase
SAM: S-adenosylmethionine
DFMA: DL-α-(Difluoromethyl) arginine
RNA: Ribonucleic acid
cDNA: Complementary deoxyribonucleic acid
RNA-seq: Ribonucleic acid sequencing
FPKM: Fragments per kilobase of exon per million mapped reads
PCA: Principal component analysis
DEGs: Differentially expressed genes
KEGG: Kyoto encyclopedia of genes and genomes
qRT-PCR: Quantitativereal-time PCR
WGCNA: Weighted gene coexpression correlation network analysis
bp: Base pair
