# Supplementary material for: BrrICE1.1 is associated with putrescine synthesis through regulation of the arginine decarboxylase gene in freezing tolerance of turnip (Brassica rapa var. rapa)
Source: BMC Plant Biol. 2020 Nov 4;20:504. doi: 10.1186/s12870-020-02697-6 (PMC7641815; doi:10.1186/s12870-020-02697-6)
Supplement: Supplementary file 1 — Additional file 1: Figure S1. The decision of power value. a The horizontal axis represents different power values. b The average network connectivity under different power values. c Network heatmap, the gene expression profile of the entire module. [file 12870_2020_2697_MOESM1_ESM.docx]

**Supporting Information**

BrrICE1.1 is associated with putrescine synthesis through regulation of the arginine decarboxylase gene in freezing tolerance of turnip (*Brassica rapa* var. *rapa*)

Xin Yin^1,2,3,4,a^, Yunqiang Yang^1,2,3,a^, Yanqiu Lv^5^, Yan Li^1,2,3,4^, Danni Yang^1,2,3,4^, Yanling Yue^6^, Yongping Yang^1,2,3,*^

^1^Key Laboratory for Plant Diversity and Biogeography of East Asia, Kunming Institute of Botany, Chinese Academy of Science, Kunming, 650204, China

^2^Plant Germplasm and Genomics Center, Kunming Institute of Botany, Chinese Academy of Sciences, Kunming 650201, China

^3^Institute of Tibetan Plateau Research at Kunming, Kunming Institute of Botany, Chinese Academy of Sciences, Kunming, 650201, China

^4^University of Chinese Academy of Sciences, Beijing, 100049, China

^5^Changchun Normal University, Changchun, 130032, China

^6^College of Landscape and Horticulture, Yunnan Agricultural University, Kunming 650201, China

^a^These authors contributed equally to this work.

^*^Corresponding author:

Yongping Yang

Email: yangyp@mail.kib.ac.cn

Phone: 86-871-65223398

**Supplementary Figures**


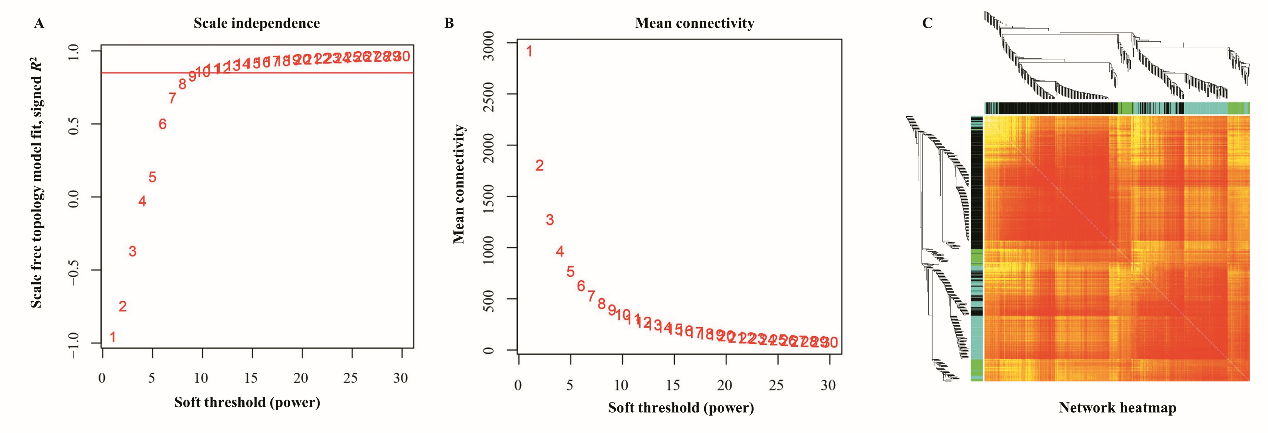


**Fig. S1** **The decision of power value.** **a** The horizontal axis represents different power values. **b** The average network connectivity under different power values. **c** Network heatmap, the gene expression profile of the entire module.


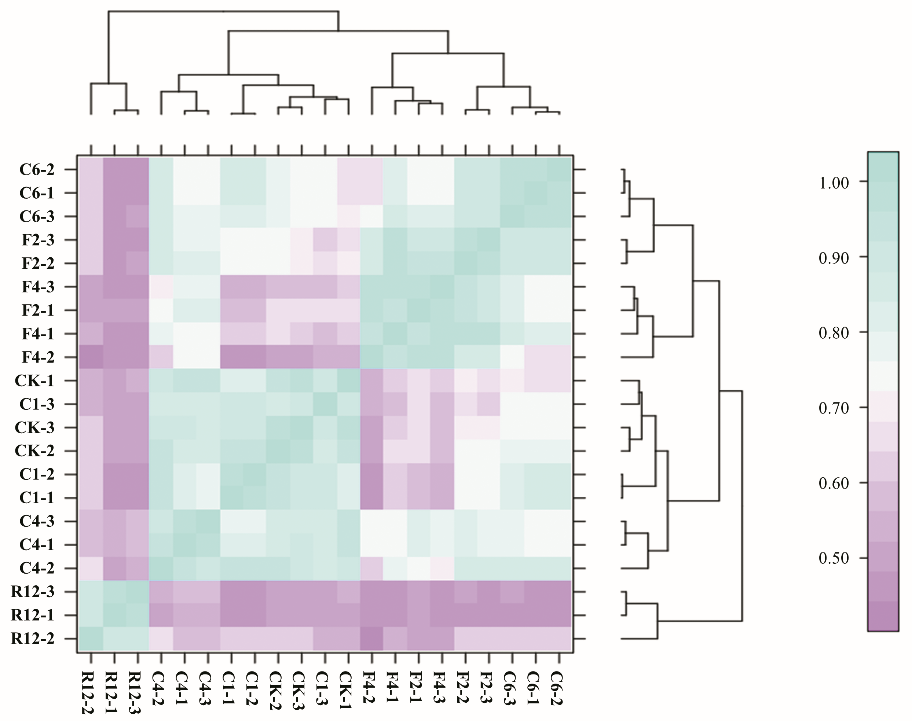


**Fig. S2** **Correlation analysis of DEGs among samples.**


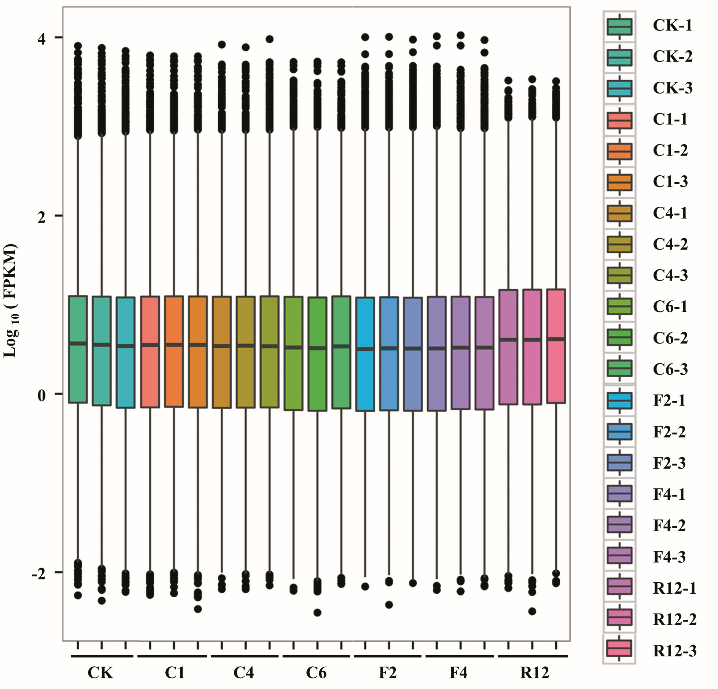


**Fig. S3 FPKM distribution for all samples.**


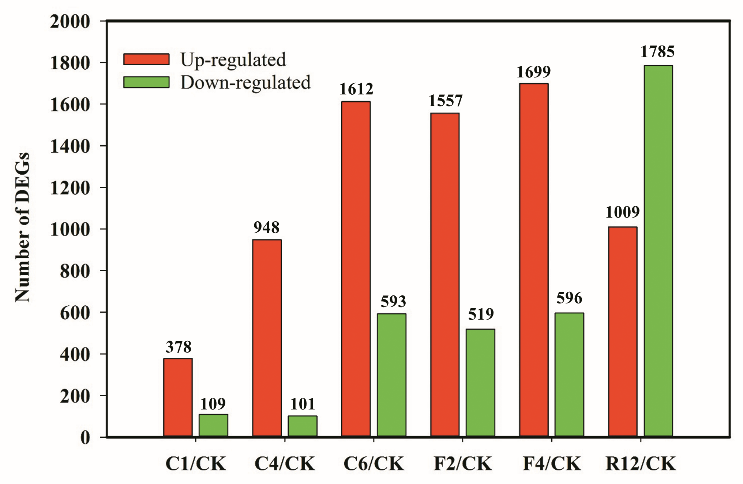


**Fig. S4** **Number of differentially expressed genes (DEGs).**


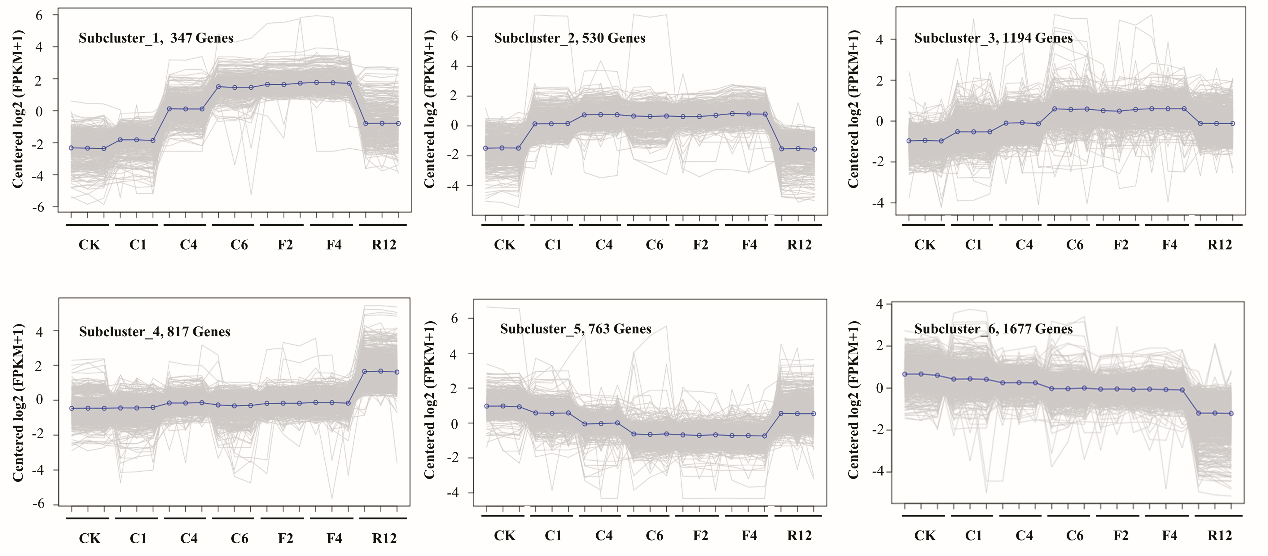


**Fig. S5 Different trend analysis of DEGs and were statistically grouped into 6 subclusters.**


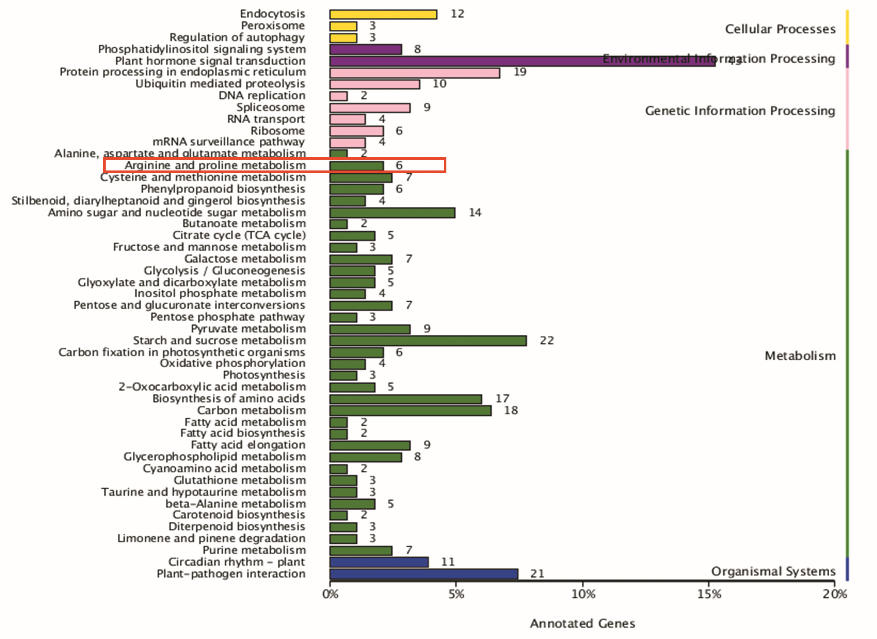


**Fig. S6 KEGG pathway analysis of module ‘Turquoise’ genes.** The red box represented for the enrichment of ‘Arginine and proline metabolism’ pathway including 6 genes.


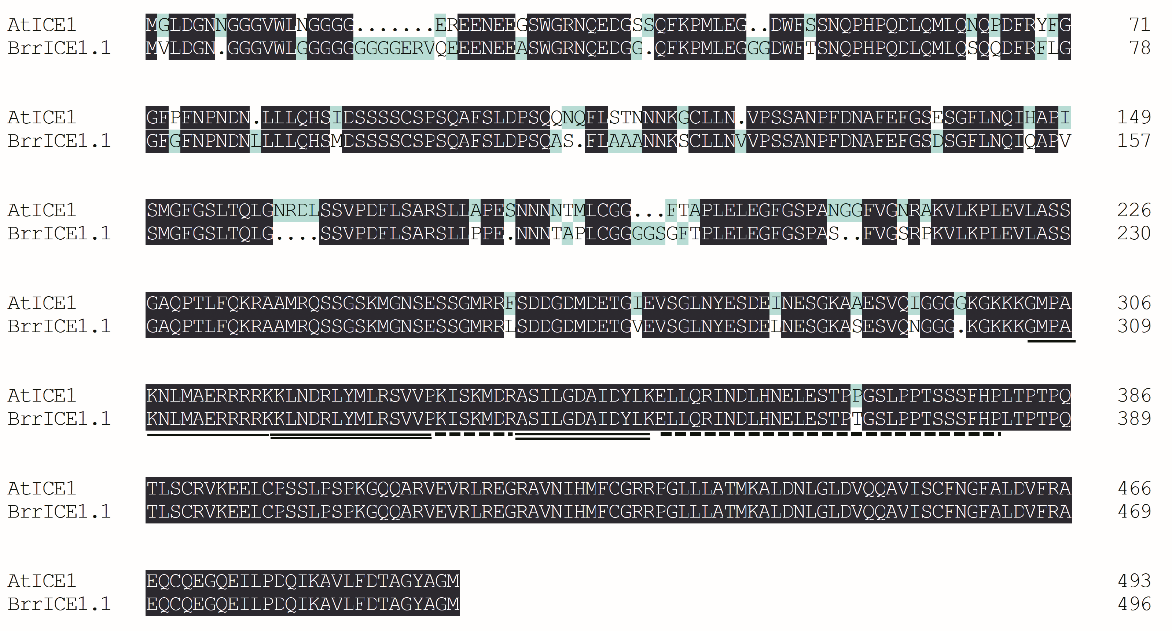


**Fig. S7 Amino acid sequence alignment of AtICE1 and BrrICE1.1.** The single bold line below the sequence indicated the basic region. Double lines represented the helix regions, which were connected by a loop, indicated by the dotted line.

**Supplementary Tables**

**Table S1. The primers used in this study.**

| Gene name | Forward primer (5’-3’) | | Reverse primer (5’-3’) |
| --- | --- | --- | --- |
| **The qRT-PCR primers for gene expression analysis.** | | | |
| *BrrADC1*  *BrrADC2.1* | CGTTGGAACTCCTCCCTCTC  CGGGGTCTTGTCGGATTTGA | | TGCAATCCCAAACCGCCTAA  GAGAGACCCTGACAACGCTT |
| *BrrADC2.2* | CTCACCAACCGAAGCCTGAT | | GCTCGATGCTCAAAACACCC |
| *BrrAIH1.1* | ACCATCAAAGCTTAGCTCCCT | | ATGGATGCTGCCTCCTTCAA |
| *BrrAIH1.2* | TGTGCAAGCCCCTCTCAGT | | CCAGCTATGTTTCGGTTAATAGCAC |
| *BrrSAMDC1.1* | GTCTACTCCAACCTCCGTTTCT | | GTCAGAGCACGGAGACCC |
| *BrrSPDS1.1* | AAAACAACCCTGCAAAAACCCT | | ATCCCCGTTCTCTGTCTCCA |
| *BrrSPDS2.1*  *BrrICE1.1*  *BrrCBF3*  *BrrCOR15A* | GATGATAACAACAACACCGCCG  CCTTATGCGGAGGAGGTGGAA  TGGCTTCTCAGGTTGAGGTT  CTCAGGAGCTGTTCTCAGTGG | | CATACGTCGCAGACTGAAAAACA  CTCTGACGCATAGCTGCACG  CATCCACTCCTCGTCCATGT  ATCAACGACTTCTTGCGCTG |
| **The primers for yeast one-hybrid verification analysis.** | | |  |
| *BrrICE1.1*  GATTACGCTCATATGATGGTTCTCGACGGAAACGG | | | GAGCTCGATGGATCCTCATACCAGCGTATCCCG |
| *BrrADC1* | TGAATTGAAAAGCTTCTTAATCAAACTATCAGAAT | | AGATCCCCGGGTACCCACGACGGAGAACAAAAAAAAC |
| *BrrADC2.1* | TGAATTGAAAAGCTTGGCTCCTGCTGAGACCATGC | | AGATCCCCGGGTACCACGTTTTCCAAATTAATGAA |
| *BrrADC2.2* | TGAATTGAAAAGCTTACAATTTATTTACATGTTTCC | | AGATCCCCGGGTACCCTTTAGAGAGAGATTGAAAGG |
| *BrrAIH1.1* | TGAATTGAAAAGCTTCATGTGAACTTAGGGCTTCC | | AGATCCCCGGGTACCATCTAATTAATCCAGAGCA |
| *BrrAIH1.2* | TGAATTGAAAAGCTTTGGTCTTCCTTTTAATTTC | | AGATCCCCGGGTACCTCCCCCCAGGTAATCTCTGTG |
| *BrrSAMDC1.1* | TGAATTGAAAAGCTTATGATGGAATCTAAAGGTGG | | AGATCCCCGGGTACCGATTCCCTCGTCCTTCT |
| *BrrSPDS2.1* | TGAATTGAAAAGCTTATGGATGCCACCAGAGAAGCC | | AGATCCCCGGGTACCATTGGTTTTTGACTCAATCACC |
| **The primers for transient expression assays in *N. benthamiana* leaves.** | | |  |
| *BrrICE1.1* CTGTTGATACATATGATGGTTCTCGACGGAAACGG  *BrrADC2.1* GCCAGTGCCAAGCTTGTGTCGCTTCTAGGAGTTTA  *BrrADC2.2* GCCAGTGCCAAGCTTAATTTGCAAACTGCTGACAA  *BrrAIH1.1* GCCAGTGCCAAGCTTTCAAGCCCATGTGAACTTAG  *BrrAIH1.2*  GCCAGTGCCAAGCTTCTTCCAAAGAGGTAAGCC | | | TCATACCAGCGTATCCCGATCATCGATGAATTC  GTCTTCCATGTCGACCTGTTTTATAAATTAC  GTCTTCCATGTCGACCTTTAGAGAGAGATTGAAAGG  GTCTTCCATGTCGACTCTCAGATTATCTCTGCGTG  GTCTTCCATGTCGACTCCCCCCAGGTAATCTCTG |
| **The primers for *Agrobacterium rhizogenes*-mediated transformation.** | | |  |
| *BrrICE1.1* (OE) ACTGTTGATACATATGATGGTTCTCGACGGAAACG  *BrrICE1.1* (RNAi) CTGTTGATACATATGTCAGATCATACCAGCGTATCCCG  *BrrADC2.2* (OE) CTGTTGATACATATGATGGTTCTCGACGGAAACGG  *BrrADC2.2* (RNAi) CTGTTGATACATATGTTACTCACTGATGCCCTCCTC | | | ATCATCGATGAATTCGATCATACCAGCGTATCCCG  ATCATCGATGAATTCATGGTTCTCGACGGAAACGG  TCATACCAGCGTATCCCGATCATCGATGAATTC  ATCATCGATGAATTCATGCCTGCTGTAGCGTGCGTCG |
| **The primers for CHIP-qPCR.** | | |  |
| *BrrADC2.2*  *BrrADC2.2-GD*  *BrrCBF3*  *BrrCBF3*-GD | | CCCGTACGCACGTTTAAA  CTCACCAACCGAAGCCTGAT  TCCAAACAATATTTTTAAGTAG  TGGCTTCTCAGGTTGAGGTT | TCTTTTTGGGGTCAAAGT  GCTCGATGCTCAAAACACCC  ATACTAGAAACAAAATCACAC  CATCCACTCCTCGTCCATGT |

**Table S2. The statistics of RNA-Seq data.**

| **Samples** | **Clean reads** | **Clean bases** | **GC Content** | **%≥Q30** |
| --- | --- | --- | --- | --- |
| CK-1 | 20,934,569 | 6,280,370,700 | 47.98% | 90.94% |
| CK-2 | 23,985,960 | 7,195,788,000 | 48.05% | 91.01% |
| CK-3 | 25,643,862 | 7,693,158,600 | 48.11% | 90.27% |
| C1-1 | 26,598,591 | 7,930,786,220 | 48.12% | 94.49% |
| C1-2 | 25,185,286 | 7,521,830,968 | 48.09% | 94.89% |
| C1-3 | 27,499,310 | 8,202,608,882 | 48.09% | 94.61% |
| C4-1 | 21,357,711 | 6,407,313,300 | 47.70% | 92.36% |
| C4-2 | 23,632,835 | 7,089,850,500 | 47.68% | 90.36% |
| C4-3 | 22,287,030 | 6,686,109,000 | 47.90% | 91.68% |
| C6-1 | 21,441,916 | 6,402,093,004 | 48.08% | 94.72% |
| C6-2 | 23,719,867 | 7,084,647,488 | 48.05% | 94.57% |
| C6-3 | 21,522,358 | 6,435,732,038 | 47.94% | 94.03% |
| F2-1 | 22,381,037 | 6,714,311,100 | 47.78% | 91.16% |
| F2-2 | 20,883,744 | 6,265,123,200 | 47.85% | 89.33% |
| F2-3 | 21,406,302 | 6,421,890,600 | 47.65% | 89.03% |
| F4-1 | 22,850,524 | 6,855,157,200 | 47.61% | 91.43% |
| F4-2 | 20,592,800 | 6,177,840,000 | 47.62% | 90.99% |
| F4-3 | 22,312,738 | 6,693,821,400 | 47.47% | 92.19% |
| R12-1 | 23,317,187 | 6,995,156,100 | 47.32% | 90.42% |
| R12-2 | 22,524,213 | 6,757,263,900 | 47.39% | 91.91% |
| R12-3 | 21,152,924 | 6,345,877,200 | 47.56% | 90.97% |

**Table S3. Summary statistics of map to reference genome database.**

| **Samples** | **Total Reads** | **Mapped Reads (%)** | **Uniq Mapped Reads (%)** | **Multiple Map Reads (%)** |
| --- | --- | --- | --- | --- |
| CK-1 | 41,869,138 | 36,126,267 (86.28%) | 31,474,398 (75.17%) | 4,651,869 (11.11%) |
| CK-2 | 47,971,920 | 41,377,812 (86.25%) | 36,032,158 (75.11%) | 5,345,654 (11.14%) |
| CK-3 | 51,287,724 | 43,815,000 (85.43%) | 38,167,017 (74.42%) | 5,647,983 (11.01%) |
| C1-1 | 53,197,182 | 47,605,358 (89.49%) | 41,588,198 (78.18%) | 6,017,160 (11.31%) |
| C1-2 | 50,370,572 | 45,190,459 (89.72%) | 39,512,943 (78.44%) | 5,677,516 (11.27%) |
| C1-3 | 54,998,620 | 49,284,669 (89.61%) | 43,101,888 (78.37%) | 6,182,781 (11.24%) |
| C4-1 | 42,715,422 | 37,167,843 (87.01%) | 32,456,310 (75.98%) | 4,711,533 (11.03%) |
| C4-2 | 47,265,670 | 40,432,353 (85.54%) | 35,329,080 (74.75%) | 5,103,273 (10.80%) |
| C4-3 | 44,574,060 | 38,439,426 (86.24%) | 33,420,994 (74.98%) | 5,018,432 (11.26%) |
| C6-1 | 42,883,832 | 38,279,322 (89.26%) | 33,616,421 (78.39%) | 4,662,901 (10.87%) |
| C6-2 | 47,439,734 | 42,305,309 (89.18%) | 37,209,946 (78.44%) | 5,095,363 (10.74%) |
| C6-3 | 43,044,716 | 38,274,677 (88.92%) | 33,678,475 (78.24%) | 4,596,202 (10.68%) |
| F2-1 | 44,762,074 | 38,508,741 (86.03%) | 33,770,592 (75.44%) | 4,738,149 (10.59%) |
| F2-2 | 41,767,488 | 35,570,492 (85.16%) | 31,250,843 (74.82%) | 4,319,649 (10.34%) |
| F2-3 | 42,812,604 | 36,280,388 (84.74%) | 31,880,948 (74.47%) | 4,399,440 (10.28%) |
| F4-1 | 45,701,048 | 39,269,164 (85.93%) | 34,399,313 (75.27%) | 4,869,851 (10.66%) |
| F4-2 | 41,185,600 | 35,254,659 (85.60%) | 30,876,443 (74.97%) | 4,378,216 (10.63%) |
| F4-3 | 44,625,476 | 38,519,034 (86.32%) | 33,797,373 (75.74%) | 4,721,661 (10.58%) |
| R12-1 | 46,634,374 | 38,831,943 (83.27%) | 34,102,728 (73.13%) | 4,729,215 (10.14%) |
| R12-2 | 45,048,426 | 37,961,533 (84.27%) | 33,266,646 (73.85%) | 4,694,887 (10.42%) |
| R12-3 | 42,305,848 | 35,312,557 (83.47%) | 31,023,200 (73.33%) | 4,289,357 (10.14%) |

**Table S4. Annotated number of DEGs.**

| **DEG Set** | **Total** | **COG** | **GO** | **KEGG** | **KOG** | **NR** | **Pfam** | **Swiss-Prot** | **eggNOG** |
| --- | --- | --- | --- | --- | --- | --- | --- | --- | --- |
| C1/CK | 483 | 133 | 431 | 148 | 198 | 481 | 381 | 366 | 440 |
| C4/CK | 1041 | 344 | 925 | 305 | 440 | 1,035 | 855 | 820 | 962 |
| C6/CK | 2179 | 740 | 1,920 | 665 | 1,010 | 2,159 | 1,762 | 1,680 | 2,028 |
| F2/CK | 2055 | 700 | 1,817 | 610 | 918 | 2,043 | 1,661 | 1,589 | 1,918 |
| F4/CK | 2272 | 787 | 2,022 | 689 | 1,054 | 2,262 | 1,849 | 1,771 | 2,117 |
| R12/CK | 2775 | 1,252 | 2,576 | 1,032 | 1,342 | 2,768 | 2,423 | 2,308 | 2,643 |
|  |  |  |  |  |  |  |  |  |  |

**Table S5. Promoter sequences of *BrrADC1*, *BrrADC2.1*, *BrrAIH1.1*, *BrrAIH1.2*, *BrrSAMDC1.1* and *BrrSPD2.1*.**

| **>BrrADC1 Promoter**  GTGTTTTTATGCAAACTACTTATAAATTGAAACGGAGAAAGTATTGTCATAATTTTATATTCTTTCGGTTTCGTAAAAAAAATATCACTTATCCAATAAAACGGCACACGTTTCATAACCTGCCGGCGACTGCTACCCAAGTGTTTGTGACGAATAGAGAATCTTTTTCTATAAAAAGTACTATAACAAAACCCCAAACCTATATTTTATCATAATTTTTGCACCCTTTTTTCATAAAAATATCATTTAGATACATTTTACGCAGATTAAAAATAATAGAATATACAATTATTAAAATAAAACGATTTAATTAAATATTTATTGATTATTTAAAAAATAAAAAATAACATTAAAATTATATTAAAAATGTAGAATAACACTTTTTTGAAACAAAATAAAAATCTAGAATAATTTTTTTATGAAACAAATGAATTATTTTTTTAATGTCTAGCTATTACAGTCTATAAAATGTTAGATGATTCTAAAATTGACAATTTTATATGTCTTATCCACCACGCCTGACGGTATTTTTCAAGGTTCTAAAAGTTGGTCTAGACGGCGCCTAAGCGGTAACTCGATTATATCCGATCGATTTAAAAAAAATCTGATTTATACTTATTTATATGATTAAAATTGGTTTAAACTTTCTAAATCGATTAAAATTGATCTAATTTTTTTCTAAATATATTAAATTAAACAATAATATTATTATAAATATACAAATTTGTCTAATTTTTTTTATTTGTATATCTAATTTTTATTCATCACAATAATTTCATAATTAAACTTAAAAACTAAAATATGTCATAGAAATGTATAATATAGATAAAATAAATCAATAATTTGCTAACGATTAGGTTTCGGCTAGGCCCCAGATAATTCGCCTAATCACTAATCTTTCTATAAAACGTAGTTACCTCCTAGCGATCTTCAGAACATTGACATTTCTTATAACATGCTGTAATATTTTGTAACTATTTTCTCTATAATATGCATAACTTACATTCTTCGAAATTTTTACTACAAACCGTATAATATAAAAACATTAATAAACTCTTAATCAAACTATCAGAATTAAGGATTTTCACAAAATAATTTATATTTATATATTTTATCAAGAATTTTATATTTATATCTCAAAATGCAGAATTTGGAAAAGGAAACATGTGATGCCAAGTGTAGAGGAAATATATTTAGAAAACTAAACGTTGCCAGGTAATGTCAAAAAAATATAGACCAATAGAACAGGAAACCATTTACTTTCTTTTTCAATTTAACACACGTATGAATTTCAGTCCACATGTCGAAAAAAGAATAACTCTAACATAAATGAGTAAGATACATATTCTTATAAAATGATAATTTCCTTTTCATTTATTTTGAAAAATTCCCCAAATTATGGTATTTTTAGACCTGGAATATATTTTGATGACAAATTGCTAAACCGAATCTATGGCATAGGAAACTAATCAAGAATAAGATTATTCTTCGTTTTTTTCTTTCTTTTATGCGGTGTAAAAGTAATAAAAAGGCGTTGAAAAACATGAAAAGTTTTTAAAGCTGATATCATAATGGGCCCTACGGCCCACTTGCCTCCTTCCTTCGTAACCTCCTCCTCCTCCACCTATATAAACAAGAGCAAGCTCCCTTCATCAATCTCACGCCTCCCTTTCATCAATCTCTATTTTCTTACGCATTCTCCCGAGAAACCTCACATTCTCTTCCTCCTCCTCAAAACCCGGATCTCTGCATGATAAGGGAATAAAACTCGTTCTTGATTTCCCTTTTTTTCTTTCCTTTAATTTTCAAGTTTTCACCTGAAACATTCACCGTGGAGATTCTGGCATCGGGTCGGGGCGCAAGAGGTGGGTTTCACGGCGGAGACAGATCCATCTTCCCCCTTCTTCTGAGGGGATAGCCGAGGCTCCGGCCTCGGCGGTTTTCAAGCCCCTACCTTTACAAATTCCCAGATTCAATAGTTTTTTTTGTTCTCCGTCGTG |
| --- |
| **>BrrADC2.1 Promoter**  GAATATTGTGAAGAGATCATACTCATGGGGCCACCAAGGTGATTGACACACTCTACAAACTTGTCATGGAGATCTGTCGTCCACTTGATCCTTGTTTTAATGGGAGATGAGCTTGTGAACTCAGAGGCCAATGAAGACCCCGCTGGCTCCTGCTGAGACCATGCATGAACCTTAGTTACTTACAATTCATTCTCTATATATATACAACTTATAACAAGTTCAGTAATTCATTTAACGATGGATGAAAAAATTTATACCGTTGAAATATCAGTTTCCACACACTCCGAAGATTGCTTTGAAAGCGAAGTGGTCTGTCTCGTTTCGGTCGAAAATATTTCGGAGATATCGTAGCATTTGCAGCAATGAGTAGCTTGTTCACTGCACATTTCATGCGAAGGATCATTATCCGATGCATGTCTTCTTCCAAACTTCATTCTACCTCTAGTTTTTTATATAAAAATGGTGATACTACGTTAAGCGTATAACATAACTGCTGGATCTAAGATGTGTTTATCTTATCGGTATTAACATCTTAAAGAAAAAAAAAAACAGTTTTCTCAAAAAAAAAAAAGAAAAAAAAACAGTCTCCAAGTGATGTGAAATACTATAGAGCTTCGCCGCGAAATTATAGACGAGTTATAACTTATAGTAAAAAAAACTATTTTTATCAAATGAGTTATAATAATTGAGTGGTCGTATAAGAAAGTATTGATTATCTCGAATGTCGACATTGTGTCACACCAATGGTGAAATTTTCATAAAGATTTCCTGACACTTCTATTACGTGTGTTAGTCAATAGTCACCTCCTTATATAATATCACCCAAAAAATAACGGGAGATATTCAAAACTATCATAAAAAACAATATTCTAACACTACCAATTAGCCATGCATGTATAATGCCAATAGACGACAAAAAGGCAGAATGGCATTGAAATACAAGTTGAATTTAACATAACTAACTCTGCACTAAAAAAATTCTTAAAAGAGTTTTTTGGATTAGTTATTTTCATTAATTTGGAAAACGTTTTTTAAAGAAAATTTAACTTTGCTGTGACTCGTGTTATGATTTTGAGATAATAATTAATTTAAATTAAAGTTTATTTAATATTAATTCATTTTCTTTTTATAATGACCTGAGTACTGATTGCATTTTAACAAAGTTGAGTTCTTTTATTTTTTTTTAGAATATAAAGCTCAACTATTTTTGAAAATTGTTGTCAACAAATACAGTAATACAGTAAAACAGATATAAAATAATACTTGATAAATTAATAATTTTTATAAATTAATAAATTTCATCGGTCTCAATTTGGACCGGTTCAAAATTTGACACTAATCGATAAATAATAACACAATATGTTAAAAAAATTTCTATGTAAATATATAACTCCATTAAAATTATAAATTAATAATTTATATATTTTTTATATAAGTAAAAACTTATTATTATGTTGTTTATTTTATATTCACAATGAAATTATCTTTATATTTTCTTAACAATTATAAAATTTTGATGAGATTTAGTAATATTATATCTAAAACCACGTTAAGTTCTACGCATATGTATTATATACCAAATAATATATTAAAATTAATATAACTGTGAAATTTTAAAAAATAACGATTAATGTTTATAAACTAAAATCAAATATTTATTATCTTAGAATAAATATATTTTAAAATAAAAAAATCTAAATAAGAAATTTTTTGTAAATTAATACCTCTATAAATTAATAAAATTTAAAAATCTCAACATTCTTAATTTATAGAGATTCTATTGTATATAACTTTCATTGCTTCCCGGTTCCGATGGGCTCTTTTTCCTGACGGGCTCAAAATTATTTGCTTCATATAATTCTCCAAAATGCAATCTTACCAATATAAATCAGGCCTGGGGAAAATGAGCTTGTTTTTAATCATAAAAATTCAAATTTATTTACGGCCTTTTTCGAAAACAAGTTTAAGATGGACCTTTTTCTTTGTAATTTATAAAACAG |
| **>BrrADC2.2 Promoter**  TCTAGTTCTCCTACTTGCAGAGTGAGATTCTCCGGTATTGATTGAAACAATAAAGCCAGTGCCATATCGTTCTTCTCATCATTACTCAGCTCTGTTTCTACTGCTTCCCAAACCTTGTGAACACGCAGGGTGATTTTCATTCTTACCGCCCACACGGTGTAGTTTGTTGTGCTCAACATTGGGCACTTAATAGATGAAGGTCCTCCGCCTCCTTTTATCTTCTCGGTCACAGCAACGATGTCACTCATGATTGATCGAAGCTCTGATACCACGTGTAGTTTTGTATAACTCGAACACAAGTTAATCACCAAACACTTCTTATTAAATTGTCTTAAGGAAACTTACTCAAAACCTTAAGCTCTCACTCACAAACTCTTAAGTTATATCATAAGCTGATATCTCCTTATATAAACTTTAATTATCCTAAACTCATTAGGATAAACACAACTCAATATTTCCTAAACTCATTAGGATATCTATTACTTGTAGTTTAAGTAACTTCAAGTTTATCCAACAGTCGACGCGTTTTCTGTTAACTTATTCTAATGCATATTCCAACTCAATATCGGATTTATCAACGTCCGGTTCAGCTGCTCTTGGATTTGGAAGTTGGAATCTGTTTTGCTTTTTTTTTAATTAAGTTGTACGTTACTAAAGAAAAAATGTACTAGTGTTACTTTTTGGTGAAGCTATTCATTCATACCCACGGTAAATAAATTGCACGTATAGACTATAGACACGAGAGAAGAACAAAAAAATTAATAGAGCTACAGAAAGTAACAAATCATAAAGAACGGGAAGACTAAACATAATCAATTTTGTAAATTTTATATTTTCTGACATGGGTTTAACTTAATTTGCAAACTGCTGACAACAATTTATTTACATGTTTCCAAATAAAAGCGGCCCGCTTACACTGTAGACTGCAGCTGCTGATAACAATGTATTTGCATAAAGCATGGGCCCGTACGCACGTTTAAAACATAGTTATCAAATTAAATTTATTTGTGACCACTAATATTTTATGTCATTTCATTCATTTGTTAACACTATTATTTATATATAGAAAATTAAAACTTTGACCCCAAAAAGACATAAATAAAATATAAAATGCATACTAATACTAAAGTAGTCTTCATAAATTTAAATATAAAATAATGTAACAAGAAAATAAATGTCACGAATTGCCATTTAAAAAATTAAATTTAAATATAAAATAATATAAAATAATATAAAAAATGCATACTATTGGGTATGTAAAACTATTTCCATGGATGCTTATAATAATATATAGAAAGGTGTATATAATAAAAACCAAAAAAATAAGAAAAAAATGTTTTCGTTTTTAAAAGAAGACTGAGAATCTTGAATCGATCAAGAATTGCCATTTATAAATATTTTAATAAAGAAGATTAGCCAAATTATGTTTTGTATTTGATGGAACTAGGTTTATTGATTGGCCCGGTTCACTGGTTTTGCCAAGTTTTTACCAGGTTTATCTTTTAATAATAATTTATTTTTATATTTTTTGCAGAAATTTGCTATGTTTACACCGGCAATAAGTTTTATGGATATATATAACTAAAATTTACAAAATTTAAATTGAAGAAATAGTAGCTATATTTTTTAATTGACTTTGTTGGTGATATATGCCTAGACATGTTTGGTTATAAACCACTTTTACAAAATTTAAATTGAAGAAACAGTAGCTTTATATGTTGGTGAATCAAGGAAATAAATTCTAGTGATTAAAATAAAAAAGAAGGAAATATCATAAATGGGCCCCACGGCCCAAATATCATAACCATCTTCTGTTTCTGTCTGCTTACTTTTCCCTCCCTTATATAAACAAGTAGAGTGTAATCTGCCTTCATCATCCTCATCAAGCTTCTCTTGTTCATTCAAGCTTGCTTTGACTTTTCTTCTGAAACGGTCATCGCGGCGGCGATAGATACACCTTCCGCCTCGTCGGCTCTAAACCTTTCAATCTCTCTCTAAAG |
| **>BrrAIH1.1 Promoter**  CAAATAGTTTAGCCCATAAAAATATCTAAAAACTACACATACGTTTCAAGCCCATGTGAACTTAGGGCTTCCAAGACTCTCTTCTTCTACACAAGCACATGTATAAAACCCCTCATGGTTTGATAGCAATTGTCATAAACCCTAATTCACTTCCGCCACACTTTTTACTCAGTTGCTAGCTCAACATGGGAAGCGATACCGAAGCAGAGAAGTCGAGTCAAAAGGAGGAGAAGAAGAAGGTTATCTCTCTTGCTCCTATCGCCAAACCTCTCGCCGGCAAAAAGCTCCAGAAGAGAACATTCAAACTCATTCAAAAAGGTACCAATCTTATACATGCTCACTCTCATTAGTGTTCCTTTTGCTGAGATTATTAAACTAAAACTTCATATAATATTTGTATCTGTTTGTTTCAGCTGCTGGAAACAAGTGTTTGAAGAGAGGCGTGAAGGAAGTGGTCAAGAGCATAAGACGTGGCCAAAAAGGGTTCGTTTTATTCTCTTGTTTATTATGCGCATTATATGTTAGTTTAATTAAAGCCGTGTACAATTAAGCCGATGCTCTGGATTGAAAACAATGAATGTAGCATGGTACTGTACACGAAAACTGAACTATGGATCGACATGTCTGCCATCTCTGAGTTCACTTTGATTAGACGGCCTGGTTTAGATCCACATTGTATCCCTCTGTTATTGTTATTAGCTGATTGTATTTAGTATGCATCTATTCTAGGAAATAGATTGTGACGTGTACAATTAAGCCGATGCTCTGGATTAATTAGATGAAGGAAGCATGGTACTGTGCACGAAAACTGAGCTTGCTGCGGATTGACATGTCTGCCCTCTTTGAGTTTGTCTTTGATAAGAGGGCCTGGTCGAGATCCACATTCTATTCTCTGTTATTGTTTTGTTTATTAGTTGTCTTAGCAATTTACTGTTTGTGGAATCCGTGTACAATTAAGCCGATGCTCTGGATTAAACATAATGAATGTAGCATGGTACTGTACACGGAAACTGAATCATGAGTTTGAAATGTCTGTGCCCTCTCTGAGTACCTAATCGGTTCTTTGATTTGCGGGCCTGGTTTAAACCCACATTCTCTTGTATATTTTTAGCTGAAAAATGATTACTTGTTGCGTTGATGCTCTACTGTGTTATTCCTCTTATTTGTATTGTAATAGATTTTTTATGTTTTTGTAGACTATGTGTTATAGCTGGAAACATTTCTCCCATTGATGTGATTACCCATCTCCCAGTCTTGTGTGAAGAAGCTGGTGTTCCTTACGTCTACGTTCCATCCAAAGAAGTAAGCCAATGTTCTTAAATTCTAAATCTATCTTCTCATTGGTCTTCTTGCTCGTGTCTAAATTTGTATGATGTTATAATTAGGATCTTGCGCAAGCCGGGTCTACGAAACGACCAACATGTTGTGTATTGGTCATGCTTAAACCGGCCAAGGGAGAGCTAAGGGCAGAAGATCTTGAAAAGTTGAAGACAGACTACGAACAAGTCTCGGAAGATGTTAAAGAGCTTTCCACTTCAGTAATCTGATTAAGAACTAGAATCAAAGATTTTGTGCCTTGGTGGCTTTTTGGTTATTGAATATGACGAGATTAAGGCGCTTATAACGATTGTAATTTAGGATCATAAAACAATGTTTTTTTATCAGTGATATTTTACTGATGGGCTACTAAATCTCTTCATTAATTACATTTCTAACAGCTTGAAATGTCAAAACAGTAATTTATTCAGACAAGTTCGTACAGGATGGGCGGAGATTCTTATTCTGATTGCCAGCTGTCAGTAAGTAAGGTCTAGATTCCGATTCCAGTAGAGGTGTGGGGTCGGGAATTGAGAGTTGAATAGTAAAGTACTGGGGTCCAATTAAAAGAAGACAATAAAACTATGTGGGTCTCCTAATAAAACCTTCACAAGTAGAGCATCACAGTTAAAATATCTGACTTGTCAGCTCTCTCCCACCTATCACGCAGAGATAATCTGAGA |
| **>BrrAIH1.2 Promoter**  TTTATTATCACTATCCAACTGTGTGTTTTAATACCTCTCTTTGATTTGTCACAGCTGCTGGAAAGAAATGTTTGAAGAGAGGCGTAAAGGAAGTGGTTAAGAGCATAAAACGTGGCCAAAAAGGGTTAGTGTGTTACATTTTATTGCAATGTTTTTGTGTGTCTTGAATGATACGTGTACAAATAAGCCGATGCTCTGGATTAAATAAAATGAATGTAGCATGGTACTGTACACGAAAATTGAATTATGGATCGACATGTCTGCCATCTCTGATTTAATATATGATTAGACGGCCTGGTTTAGATCCACATTTAATATCTTTGTGACCATCTCTAGTATGCATATGTCTGTATAATACATTGTAATTGCACCTATACGTGTACAATTAAGCCGATGCTCTGGATATTTAATAGAATGAAGGTAGCATGGTACTGTGCACGTAAACTAAAATCGGATCGACATGTCTGCCATCTCTGATTCATGTTGTGATGAGACGGCCTGGTTTAGATCCACATTCTTGTCTTTTGTTTAGTACTTGAGATCATTAGTTGAACATCAGTGGTTGAGTTCATTAATATAATTGTTTGTTTGCGTAGAGTTAAGTTTCACATTTTAGTTTTTTTTTCTAATTTAAATTCGTGTACAATTAAGCCGATGCTCTGGATGAACTAGATGAAGGTAGCATGGTACTGTGCACGTAAACTGAGCTTGCTTGCGGATTGATATGTCTGCCCTCTATGAGTTTTTCTTTGATTAGAGGGCCTGGTTGAGATCCACATCCTATGTTTTTGCTTGTGTTTTGGTTTGTTTTTTATGTATATGTGTGATCCGTGTACAATTAAGCCGATGCTCTGGATTAAATATAATGAATGTAGCATGGTACTGTCCACGGAAACTGAATCATGGGTTTGAAATGTCTGTGCCCTCTTTGAGAACCTAACTGGTTCTTTGATTTGCGGGCCTGGTTTAAACCCACATTCTCTTTGTCTAAATTTTGCATTAAAATTGATTACTTGTTTCCTTTAATGCCACTCTTTAATCTTCTGCCTTGTGTTCTAATTGAGTGTTCTTTGTTATGTAGAATATGTGTTATTGCTGGAAACATATCTCCGATTGATGTGATTACCCATCTTCCAATCTTGTGTGAAGAAGCTGGTGTTCCTTACCTCTACGTTCCTTCCAAAGAGGTAAGCCAATGGTCTTCCTTTTAATTTCTTGTCTCGTGTCCTATGGAGATTGTTCTTGGGTCTAAGACAGTCAAATGTTTGTTTTGTGTAACTCAGGATCTTGCGCAAGCTGGTTCTACAAAACGACCAACGTGTTGCGTCTTGGTCATGCTTAAACCGGCTAAAGGAGAGCTAAGCGCAGAAGATCTTGAAAAGCTAAAGACAGACTACGAACAAGTCGCTGACGATGTTAAAGAGCTTGCCTCTGATGTAATCTGATTAGAAACTAGAATCAAAGAGCACACTTAACATGTGTTTGTGTCGTATCTTAGTGGTTTTGTTGGTTAATGAATATGATTAGATTTGAAGGACTTGAAAAATATGCTTTTCACATTTATTCAATTCTGTTGTTTCGAATTTGTGTCGACTGCAATTCAGAACATAAATTACAATTAATAATTTTAAGACATGATAAATACGGTAATTACAGAACAGCTTAAAATGTTAAGACAAGGAAAATAAAAACATCCCAACAACTTTTGTCGTGATTCGAAACTTGGGTCGAGATTCTGTTTCTGGTTAGTAGCTACCACCAGGCCCACCACCAAGTAAAGTTCTAGATTCTGATTCTTTCACAACACTAGAGGTGGGGTCAGGTATTGGGAATGTTAAGAGTTGTACAGTAAAGTAATGGGGTCCAATTGTAAGAAAAACATAACTACGTGGGTCTGGTCTTGTTATAAAAACCTTGAATATAACTAAACAAGTACTAGCAGTTAAAATATTTGACTTTCCGCGTTCTCTCCATCACCACACAGAGATTACCTGGGGGGA  **>BrrSAMDC1.1 Promoter**  AAGGTTTGTCTTTTACTTTCTGGTTTTAATAACTCTTCTTGGGAACTTGTTAACAGATAGTGAATGAATAAGACATGACATGATTTAACTTGATGATGGATGATCGATGTTATCTCACTTCTAGTGAACAATTGATGTTGATGATTATGATTGGTAAATCTCTGAGCTTTCTTTGAGTTAACAAACAATTGTGGCATTAATCCTTAGTTTGTATTCATCATCAGGTATCAAAGCGTTTTGAAGAACTGAAACTGGATTTATGAGAATTGAGAAGACTATTACCAAACCGTAAGAAAAAGAAACGGTTTAATCTTGTAACATATACCATGTGATTTGGGATTCGAATTCTTGTACACATATCATTTTATCTATCTTGTTGGAATATATTCTTCTGTCATCATATTTGACAAGTTGGGTGGGAATATCTTATCAGAAACCCCCTCTGAATCATTCTAGCAACTTCTAGGTAAAAGAAGAAGGTATATGGTTCTGTTTTGTATGAAAATCTCACTATTACAAATGTGAAAGATAAATTATTTTGTTTGGCTGTGGGTAAATAAACTATAGAAAGTGTGTATGTCTGTACAAAATCTAGATACTTTTTCAAAAGACAATTCCACCTTTAGACTAGAATAATTACGTACTTTTCATATCAAAAGTCCAGTGTTGATTATTACAAAATTGTATAATCTTTATGTTTGTAGCTTTGTTGACTAATGGAATCATGAATGAGTCACGAGAGGGTAATGGAGAGCCTTGGCGAATAGAAAATAAAATTCGATTCTTTGGGCCGCGCTACGTGACAGCCCACCTCGAGCTATTCGCCCAGATGATATACGTGTCATTATTTAACTGGTTGGTAGTTATCTAGTAATGTTGTTGCTTGTTATTCTCGGCGGAAAGATAATGTCAAAATATTTGTTTCAAAACAATAAAATAAGTTTCCTAGTGGATACGGAAACAAACAAATAAAATAAAATAAAATAAAATGTATTAATTACTATAAGATTTGTTGCTTTTATTTTAAGCTGTGGTCAGGAATCACGCAACCTATCGTTGAAAAATAGAACAAGAAAATGAACGGCTGAGGAGAGTCCTGAGCTTTCGCCTGTTTAAGGTTAAAGGGTAAAGAAGTAAATAACCGTGGCGCAATTAGGGCTTATAAAAGAAAGTGTTTCTACGTTGGCTTTCTCTTCACGAACGCCCCTCATCATTACCTAATCATCATCTTCAACCTTCTCTCAAATTAGAGTTTTCAATTGATCAAAAGTCTCGCTCTTTCTCCCCAATCATCTGGATCTGGTGAGCTTTTTGTTTTTCTTTATTTGCGTTATAGATCTATCTGATTATTGTTCTTATCGCGAAATTGTTTTCTTAATGTAGAATTTTCGATTGTTTCGACTTTTTCAGGTTTGATATCTGTGGAAAGAAGGGATTCATAATTCGTGTTCTTGATCTAAAATTCATAAAATATGTAAGTTTTCGATTGATCTTCGCATATAGTATTGGTTTATGTATAAGAGATTGATCATTCTGAGCTATGTTGATATATTCATTGTTAGATAACAAATCGTTACGTTTTAAGGGTTGATTAGCCGTAATCTTAGGTTCATATTGTTGTATTGGTATAATGAAACAATTTAATCATGTCTCGTTTGGTAAACATTTATGCTAGGATGAGTAGTAAACGTTCTCGATTGATCTAACGCATATGCTATTGGTTTTTGTATAACAGATTGATTTTATATCTGAATTGAAATACTTATATTTTGATTAATGTTACATATACATAACTGTTTTTAAGGGTTAACTGTAATCTTAGGCTCACCATAGTTGTAGATCTGCTGCAACAATTTGATCATATGTCTCATCTAAGAATGAGTATTGAACGTTTTTTTAGATATGTTATTGAAATTTTGAAACTTTGAACATTGATGAGTATCTGTTCACCTTTACCTAACCATATGGTTTTGTTGATTACAGAACGCGTGAATGAGCTT |
| **>BrrSPDS2.1 Promoter**  ACATTATTCGCGACCACTTGCATAAAATAATATATTTTATGTGGTGTGCAATAATGTTTGGAATTATTGTAAGTTTTAATATCATATATATCAATATTATGTTCACATAATATTGAAAATAAACAAACATATTTTTTGACCAAAAAAAAACAAAAATATTTAATATTTATATATATATAAACAACGCGTTAGAATGAAATAATTTTTTTTTGGAGCTAATGTATATAATATATTATTCTAATTTTAATACGAACTATAATGTTTGAAAATCATATATATGAAGATTGTTTTGACAAAAAAACATTTTTTATTTATTAGTTTTTGCTTAAAATAAATAACACTATATCATTTGGAAAATTTTGTTATAAACTTGATATATTTAGGTTAATAAATTATGAAGAAAAATCGTGAAATTAATTATCAGAAATTCATAAGCAAGAAATATGGTGTCACTGACTCACTTGGTTGGTTCATTCGGACAAATCATACCTTATTAGTTTATTTAGCATTCAATGTCGTCAAAAAAAAAGCATTCACTAAACAAGAATGAAAGAGAAAATAATATTATTTCCCGATTCTTTTCCAATTTTGAAAATCTTAAGTATATATAACTCCTATAAGAGTGGATTTGAGTGGTTCACCTACCGCACTAAAATAGGTCATTATATCAAATGTGATTATGTGAACATGTGAAAGTATATTAGTTATATTATATCATTATAATATAGTTTAGTCAGCGTCTGTGTTTGAATTAAAATTAAGATAAACTTGGAGAAATTTGGGATTTTGGTATCAATTGCTATGACATGTGTTTATGCTAAAACTGGCTCTCAATCTTCGCCACTAGGCCCACCACGGCTAATCATGATTGGTTTAGATCCGGTCACTGACACAATCTTAGCCACATTAGACCCACAAGGATTTGTAACATTCCTTTTACTATCTTTTTAGTAATAAAGAACTCGTTTTTTTTTCATACGTGTTTTTCGTTTCTAATATCGTATTCGTAAAAGATTTCGGCTCAGCTAAAACCAAATTTAGTGGACCGTTCAAACTGAAATGCTTGGCTTCAACTAGGAAGAGACGTATGAAACTGAAACAGCAGTATTCATGGTCGGTACTTGTTATCAGACGAAATATATCTGCCTAATATACACCTCTACGAATGTTCAAAAAAAAAAAAACACCTCTACAAAAATTATTGTGTATTCGTGAAAAGGGGCTCCTTTGCATGATATTTGATTTTGCAAAAGCTGGGTTCTATAGTGGGCCTAATAATATGATAGCCTAAGCGACCCAAGTCCTGCTATCCAATTATCTTTGGACAGTATCTGTGTTACCTGGGAGACTTATGAGAGCCATATTACAAAACGATTCAAGGACATCAAATTAAATACAGTAGAAAACTATATAAAAAAATTGTAAATGAAACTTGTTATTCGGTAACTAATTTATCTTATATACTTCCCTATGTAATAGTTCAATGGTTGTGTTATCACTGGTAAATATATCACATACACATCAACATAAACACTTACAATTAACATCATAAAACTGTTTTGTTTTATGATTAGAGCCTCCTCTACAAGAATATATCTGACACTTGCTAATCCTCTCTATGTTGTGTGATTTGGCCGAGCTTCGAACCCAAGCATGCGTTGCACGTGAAATAGCGCGTGGCGTAAACGTGGTGTCTGGCAAGTGAGTATCATCAAGAAGCGAATAAGAAAAACAGACAGACCAAAAAGCCAAGACAACGATATATTCGAGGCAAAAGCAGTTTCGTCCGTATTATTTATCATCACATAATAATTTCGCAATTAACTCGTCGGAATTATAACCCACAAAATAAAATAACATAAATGAAATTGATATTTTACAGTCTTACTATCGAATCTTTTACTTCTTAATTCTTATATATTCCGCTTCACTTCACATCCAAACAAATCCTGCAAAAAAAAAACCCTAATCCCCTCTCTCGTCTTCTATCAGCAGTAGCA |

Note: The yellow mark represented MYC element (CANNTG) in promoter region.
